# Ethnicity and involuntary hospitalisation: a study of intersectional effects

**DOI:** 10.1007/s00127-025-02898-0

**Published:** 2025-04-16

**Authors:** Rooble Ali, Susan Walker, Patrick Nyikavaranda, Johnny Downs, Rashmi Patel, Mizanur Khondoker, Kamaldeep Bhui, Richard D. Hayes, Daniela Fonseca de Freitas

**Affiliations:** 1 Institute of Psychiatry, Psychology and Neuroscience, King’s College London, London, UK; 2 Division of Psychiatry, University College London, London, UK; 3 Great Ormond Street Institute of Child Health, University College London, London, UK; 4 Department of Primary Care & Public Health, Brighton & Sussex Medical School, University of Sussex, Brighton, UK; 5 South London and Maudsley NHS Foundation Trust, London, UK; 6 Department of Psychiatry, University of Cambridge, Cambridge, UK; 7 Norwich Medical School, University of East Anglia, Norwich, UK; 8 Department of Psychiatry, Nuffield Department of Primary Care Health Sciences, Wadham College, University of Oxford, Oxford, UK; 9 NHS England, London, UK

**Keywords:** Race, Detention, Intersectionality, Inequity, Disparity

## Abstract

**Purpose:**

Studies have found that the Mental Health Act is used disproportionally among minoritised ethnicities. Yet, little research has been conducted to understand how the intersectionality of ethnicity with sociodemographic factors relates to involuntary admission. This study aimed to investigate whether an association between ethnicity and involuntary hospitalisation is altered by variations in service-users’ sociodemographic positions.

**Methods:**

A retrospective cohort study using records from the South London and Maudsley identified 18,569 service-users with a first episode of hospitalisation in a 13-year period. Logistic regression was used to calculate odds ratios for involuntary hospitalisation across ethnicities while adjusting for sociodemographic (age, gender, area-level deprivation, homelessness, and migration) and clinical factors (psychiatric diagnosis and HoNOS scores). Interaction analysis was conducted to identify intersectional effects between ethnicity and sociodemographic variables, potentially modifying the odds ratios of involuntary admission across ethnic groups.

**Results:**

Increased odds of involuntary hospitalisation compared to White British service-users were observed among 10 of the 14 ethnicities, with around, or just under twice the odds observed for Asian Chinese, Black African, and Black Caribbean. Women were found to have increased odds of involuntary admission. Significant interactions were present between ethnicity and age, area-level deprivation, homelessness, and migration in the unadjusted models. These effect modifications were not significant after adjustment for confounders.

**Conclusions:**

Ethnic inequalities were observed in involuntary hospitalisation among service-users on first admission. No evidence of intersectional effects was present when adjusting for sociodemographic and clinical factors. Further research needs to identify the mechanisms causing the inequalities.

**Supplementary Information:**

The online version contains supplementary material available at 10.1007/s00127-025-02898-0.

## Introduction

### Mental health act and involuntary hospitalisation

Involuntary psychiatric hospitalisation features heavily in modern psychiatry, a medical and legal practice with the potential to infringe on civil liberties [1] and an individual’s right to self-determination [2]. Some service-users have described detainment under the Mental Health Act (MHA) as necessary to protect them at their most vulnerable [3], whilst others have described the intervention as coercive, distressing, and disempowering [4]. Clinical outcomes are also mixed, with some studies reporting positive results [5, 6], while others found worsening rates of suicide, length of stay, and rates of readmissions [7].

### Ethnicity and involuntary hospitalisation

Evidence from systematic reviews and meta-analyses show that minoritised ethnicities are at higher risk of involuntary hospitalisation when compared to their White ethnic peers, both within the UK [8–10] and internationally [11]. In the UK, studies with services-users of secondary mental healthcare with psychosis receiving outpatient and inpatient care [12, 13] and studies with only inpatient care population [14, 15] show that Black African and Black Caribbean people have over twice the odds for involuntary admission compared to White British service-users. In contrast, studies investigating Asian ethnic groups yielded mixed results, with some showing no differences [12, 16, 17], whilst others, including a meta-analysis, have found South Asian people have a 1.3 times greater risk of involuntary admission [10, 11, 14, 18]. The contrasting findings in this group may be due to differences between studies in sample sizes, adjustments, or restricting the cohort by diagnosis. Fewer studies examined involuntary hospitalisation in East Asian communities, and evidence suggests East Asian service-users have over twice as likely to be admitted involuntarly compared to White service-users [11, 18]. There is also a paucity of studies examining inequalities in individuals of mixed ethnic backgrounds, due to small sample sizes and statistical power. When mixed ethnic groups were included, common problems involved not describing the make-up of the category [19] or including an aggregated category for all mixed ethnic groups [12].

### Intersectionality

Variations in sociodemographic factors may interact with ethnicity to modify the risk of involuntary hospitalisation. Intersectionality, a term coined by Crenshaw [20], describes a multidimensional analytic framework where the experience of multiple overlapping identities of gender, race, and class contributes to power imbalances that are greater than the sum of their parts. This approach broadens an understanding of inequity, replacing a single-axis system that focuses on the most privileged group members and marginalises the multiply burdened. Despite the need for an intersectional approach to the study of ethnic inequalities being identified [9, 11], the literature remains scarce in investigating if the interaction with sociodemographic variables can influence the relationship between ethnicity and involuntary hospitalisation.

Studies focusing on the main effect of sociodemographic characteristics (i.e., without adopting an intersectional approach) show evidence of associations between many factors and involuntary hospitalisation. For example, a meta-analysis by Walker et al. [21] found that men (of all ethnicities) had a greater risk of involuntary hospitalisation than women. However, the recent meta-analysis by Barnett et al. [11] reports that study samples with a higher percentage of women had stronger associations with involuntary admission among Black Caribbean, Black unspecified and South Asian ethnicities (no interactions were tested in other ethnicities). Further, studies have found evidence of associations between age and involuntary hospitalisation, such that younger service-users had the highest risk [10, 22]. But, a recent meta-analysis showed no interactions between Black Caribbean ethnicity and age for involuntary admission, with no interactions being tested for other ethnicities [11]. Neighbourhood deprivation has been associated with compulsory admission, with some studies suggesting a dose-response relationship [10, 21]. Furthermore, migrants were also more likely to be involuntarily hospitalised when compared to the native groups [11, 23–25].

### Objectives and hypothesis

The current study investigated potential ethnic inequalities in involuntary admission in the first episode of hospitalisation and whether the intersectionality of ethnicity with age, gender, area-level deprivation, homelessness, and migration alters the associations between ethnicity and involuntary, compared to voluntary, hospitalisation. Firstly, based on previous literature, we hypothesised that minoritised ethnic groups had an increased risk of involuntary hospitalisation compared to the White British ethnic group. Secondly, we hypothesised that there is an interaction between ethnicity and key sociodemographic factors, such that minoritised ethnic service-users who are younger, male, from areas of higher deprivation, homeless, or migrants had an exacerbated risk of involuntary hospitalisation.

## Methods

### Study design and setting

This retrospective cohort study used routinely collected patient information from the South London and Maudsley National Health Service Foundation Trust (SLaM) [26]. SLaM, one of Europe’s largest secondary mental health providers, has fully electronic health records (EHRs) since 2006 and provides care for people living in the London boroughs of Lambeth, Southwark, Lewisham, and Croydon [27].

The Clinical Record Interactive Search (CRIS) system was set up in 2007 and was used to extract data sourced from EHRs in SLaM, allowing researchers access to de-identified information in structured and unstructured fields [28]. In this study, data were extracted exclusively from structured fields. CRIS dataset received approval from the Oxford C Research Ethics Committee (18/SC/0372). This project gained approval from the service-user-led CRIS oversight committee (ref. 19–066).

### Participants

The cohort inclusion criteria consisted of service-users that (a) had a first episode of voluntary or involuntary admission under the MHA that started or finished during the observation period of 01/01/2008 to 31/05/2021; (b) a personal address or be registered with a general practitioner (GP) in the SLaM catchment area within the observation period, or lived in London at the time of admission; (c) over the age of 18 at time of the first admission; (d) stayed at least one night in hospital. Individuals with missing data on ethnicity, age, or gender were excluded from the cohort at the point of entry.

### Exposure and outcome

Ethnicity was divided into self-ascribed categories following the NHS classifications. Due to small numbers, the ethnic groups of Mixed ethnicity White and Asian were merged into ‘other Mixed background’, and the categories of White Gypsy/Irish Traveller, Other ethnic group– Arab and any Other ethnic group were merged to form ‘Other ethnic background’.

The outcome of interest was involuntary hospitalisation under the MHA Sects. 2, 3, 4, or 5(2) applied within two days of the first admission to SLaM during the observation period. Involuntary hospitalisation was compared to voluntary hospitalisation.

### Demographics and clinical factors

The sociodemographic factors investigated included age, gender, area-level deprivation, homelessness, and migration. Adjustment for clinical factors comprised of psychiatric diagnoses and Health of the Nation Outcome Scale (HoNOS) items.

Area-level deprivation was calculated from the English Indices of Multiple Deprivation (IMD) and divided into quintiles [29]. The version of the IMD score recorded (2007, 2010, 2015 or 2019) was based on the service-user’s address closest to the date of admission. Homelessness was calculated as whether the individual had been homeless at the point of admission, in the previous year, or had a risk assessment mentioning unstable housing recorded 12 months before or 28 days after admission.

Migration was ascertained if: (a) a language other than English was listed as their first language; (b) an interpreter was documented as needed; (c) country of birth was not listed as the UK; (d) there was evidence of an asylum application.

Psychiatric diagnoses were assessed as per the ICD-10 categories and were recorded within 28 days prior and 28 days post-admission. The Health of the Nation Outcome Scale (HoNOS) is an instrument conceived to assess the social and physical functioning of service-users with mental illnesses [30]. The HoNOS score used was retrieved hierarchically: (a) within 28 days before admission; (b) if unavailable, then within 28 days after; (c) if unavailable, then within 12 months before admission.

### Statistical analysis

We used multivariable logistic regression to estimate odds ratios (OR) of involuntary hospitalisation among minoritised ethnicities compared to White British service-users. We estimated the OR in unadjusted models and models adjusting for sociodemographic and clinical factors. We tested statistical interaction in logistic regressions to assess the possible effects of the intersectionality of ethnicity with each of the sociodemographic factors in unadjusted and fully adjusted models. This was done using contrast analyses, which compare the model with and without the interaction term. Some variables contained missing data, namely, area-level deprivation (11.9%), migrant status (32.1%), and HoNOS items (18.2% - 21.8%). To preserve statistical power, missing data were categorised as undetermined. Data were analysed using STATA version 15 [31].

## Results

### Descriptive data

**Fig. 1:**
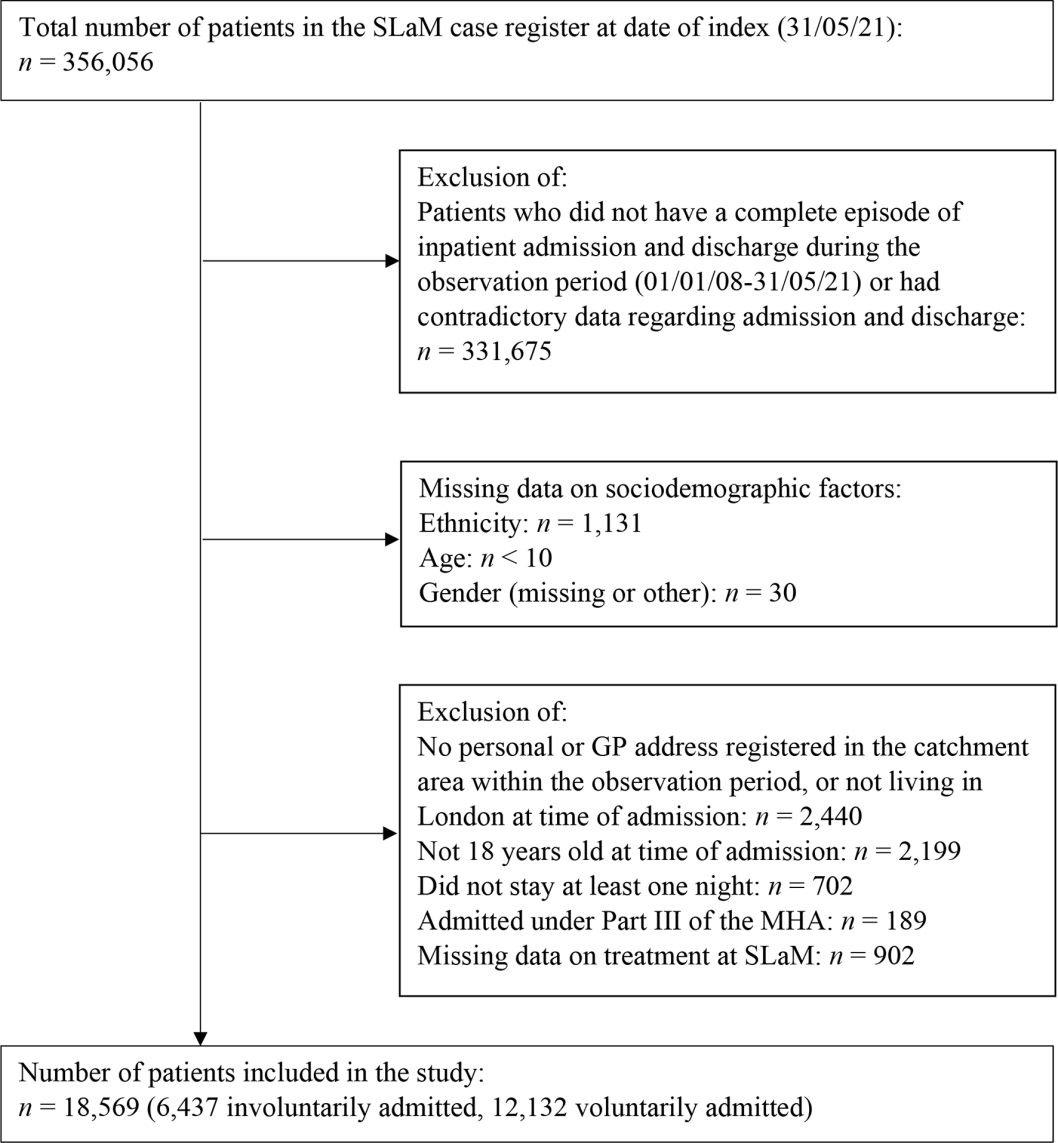
Flow diagram of study cohort selection

From a total number of 356,056 service-users with records on 31/05/2021, 18,569 service-users were identified as meeting the inclusion criteria for the study (Fig. 1). Of this cohort, 34.7% had been hospitalised involuntarily (Table 1).

The largest ethnic group in the cohort was White British (44.4%), The largest age group in the cohort was 35–49 years old (31.4%), and the sample mean age was 42 years. The gender distribution was 55.7% male. In addition, 18.9% of the cohort was classified as homeless, and 27.5% had been categorised as migrants (Table 1).

**Table 1:** Demographic and clinical characteristics of service users with a first episode of admission in slam within the observation period

	Total *N* (% of total sample) ^a^	Voluntarily admitted for inpatient care on first admission (% per characteristic)	Admitted under Sects. 2, 3, 4 or 5(2) on first admission (% per characteristic)
Total	18,569 (100)	12,132 (65.3)	6437 (34.7)
**Ethnicity**			
White British	8240 (44.4)	6,367 (77.3)	1873 (22.7)
White Irish	513 (2.8)	385 (75.1)	128 (25.0)
Other White background	1852 (10.0)	1109 (59.9)	743 (40.1)
Black African	2031 (10.9)	981 (48.3)	1050 (51.7)
Black Caribbean	1145 (6.1)	569 (49.7)	576 (50.3)
Black British / Other Black background	1887 (10.2)	966 (51.2)	921 (48.8)
Asian Bangladeshi	90 (0.5)	48 (53.3)	42 (46.7)
Asian Indian	287 (1.6)	183 (63.8)	104 (36.2)
Asian Pakistani	159 (0.9)	92 (57.9)	67 (42.1)
Asian Chinese	130 (0.7)	62 (47.7)	68 (52.3)
Asian British / Other Asian background	661 (3.6)	398 (60.2)	263 (39.8)
White and Black African	83 (0.5)	57 (68.7)	26 (31.3)
White and Black Caribbean	227 (1.2)	159 (70.0)	68 (30.0)
Other Mixed background	171 (0.9)	115 (67.3)	56 (32.8)
Other ethnic background	1093 (5.9)	641 (58.7)	452 (41.4)
**Sociodemographic variables**			
Age			
18–24	3006 (16.2)	1792 (59.6)	1214 (40.4)
25–34	4571 (24.6)	2968 (64.9)	1603 (35.1)
35–49	5835 (31.4)	4048 (69.4)	1787 (30.6)
50–64	2958 (15.9)	1932 (65.3)	1026 (34.7)
65–99	2199 (11.8)	1392 (63.3)	807 (36.7)
Gender			
Male	10,343 (55.7)	6795 (65.7)	3548 (34.3)
Female	8226 (44.3)	5337 (64.9)	2889 (35.1)
Area-level deprivation			
1st quintile (least deprived)	2437 (13.1)	1719 (70.5)	718 (29.5)
2nd quintile	3367 (18.1)	2184 (64.9)	1183 (35.1)
3rd quintile	3566 (19.2)	2304 (64.6)	1262 (35.4)
4th quintile	3607 (19.4)	2296 (63.7)	1311 (36.4)
5th quintile (most deprived)	3387 (18.2)	2171 (64.1)	1216 (35.9)
Undetermined	2205 (11.9)	1458 (66.1)	747 (33.9)
Homeless			
No	15,058 (81.1)	9278 (61.6)	5780 (38.4)
Yes	3511 (18.9)	2854 (81.3)	657 (18.7)
Migrant status			
No	7513 (40.5)	5615 (74.7)	1898 (25.3)
Yes	5099 (27.5)	2938 (57.6)	2161 (42.4)
Undetermined	5957 (32.1)	3579 (60.1)	2378 (39.9)
**Psychiatric diagnosis (ICD-10)**			
Mental disorders due to known physiological conditions (F01-F09)			
No	17,724 (95.5)	11,596 (65.4)	6128 (34.6)
Yes	845 (4.6)	536 (63.4)	309 (36.6)
Mental and behavioural disorders due to psychoactive substance use (F10-F19)			
No	15,562 (83.8)	9581 (61.6)	5981 (38.4)
Yes	3007 (16.2)	2551 (84.8)	456 (15.2)
Schizophrenia, schizotypal, delusional, and other non-mood psychotic disorders (F20-F29)			
No	14,919 (80.3)	10,632 (71.3)	4287 (28.7)
Yes	3650 (19.7)	1500 (41.1)	2150 (58.9)
Affective psychosis (F30.2, F31.2, F31.5, F32.3, F33.3)			
No	17,786 (95.8)	11,747 (66.1)	6039 (34.0)
Yes	783 (4.2)	385 (49.2)	398 (50.8)
Mood disorder (F30– F39, except the affective psychosis codes)			
No	15,737 (84.8)	9925 (63.1)	5812 (36.9)
Yes	2832 (15.3)	2207 (77.9)	625 (22.1)
Anxiety, dissociative, stress-related, somatoform, and other nonpsychotic disorders (F40-F48)			
No	17,038 (91.8)	10,892 (63.9)	6146 (36.1)
Yes	1531 (8.2)	1240 (81.0)	291 (19.0)
Behavioural syndromes associated with physiological disturbances and physical factors (F50-F59)			
No	18,313 (98.6)	11,923 (65.1)	6390 (34.9)
Yes	256 (1.4)	209 (81.6)	47 (18.4)
Disorders of adult personality and behaviour (F60-F69)			
No	17,837 (96.1)	11,540 (64.7)	6927 (35.3)
Yes	732 (3.9)	592 (80.9)	140 (19.1)
Intellectual disabilities (F70-F79)			
No	18,459 (99.4)	12,064 (65.4)	6395 (34.6)
Yes	110 (0.6)	68 (61.8)	42 (38.2)
Pervasive and specific developmental disorders (F80-F89)			
No	18,496 (99.6)	12,088 (65.4)	6408 (34.7)
Yes	73 (0.4)	44 (60.3)	29 (39.7)
Behavioural and emotional disorders with onset usually occurring in childhood and adolescence (F90-F98)			
No	18,534 (99.8)	12,108 (65.3)	6426 (34.7)
Yes	35 (0.2)	24 (68.6)	11 (31.4)
**Health of the Nation Outcome Scales (HoNOS)**			
Overactive, aggressive, disruptive, or agitated behaviour			
No problem	5763 (31.0)	4252 (73.8)	1511 (26.2)
Minor problem	3294 (17.7)	1978 (60.1)	1316 (40.0)
Mild problem	2991 (16.1)	1581 (52.9)	1410 (47.1)
Moderately severe problem	2052 (11.1)	888 (43.3)	1164 (56.7)
Severe to very severe problem	1092 (5.9)	393 (36.0)	699 (64.0)
Undetermined	3377 (18.2)	3040 (90.0)	337 (10.0)
Non-accidental self-injury			
No problem	9436 (50.8)	4872 (51.6)	4564 (48.4)
Minor problem	1744 (9.4)	1108 (63.5)	636 (36.5)
Mild problem	1552 (8.4)	1148 (74.0)	404 (26.0)
Moderately severe problem	1446 (7.8)	1153 (79.7)	293 (20.3)
Severe to very severe problem	986 (5.3)	807 (81.9)	179 (18.2)
Undetermined	3405 (18.3)	3044 (89.4)	361 (10.6)
Problem drinking or drug taking			
No problem	9186 (49.5)	5504 (59.9)	3682 (40.1)
Minor problem	1404 (7.6)	819 (58.3)	585 (41.7)
Mild problem	1765 (9.5)	1049 (59.4)	716 (40.6)
Moderately severe problem	1672 (9.0)	1038 (62.1)	634 (37.9)
Severe to very severe problem	900 (4.9)	591 (65.7)	309 (34.3)
Undetermined	3642 (19.6)	3131 (86.0)	511 (14.0)
Cognitive problems			
No problem	8694 (46.8)	5645 (64.9)	3049 (35.1)
Minor problem	2771 (14.9)	1528 (55.1)	1243 (44.9)
Mild problem	2047 (11.0)	1057 (51.6)	990 (48.4)
Moderately severe problem	1175 (6.3)	611 (52.0)	564 (48.0)
Severe to very severe problem	429 (2.3)	219 (51.1)	210 (49.0)
Undetermined	3453 (18.6)	3072 (89.0)	381 (11.0)
Physical illness or disability problems			
No problem	9140 (49.2)	5284 (57.8)	3856 (42.2)
Minor problem	2291 (12.3)	1393 (60.8)	898 (39.2)
Mild problem	1996 (10.8)	1261 (63.2)	735 (36.8)
Moderately severe problem	1230 (6.6)	817 (66.4)	413 (33.6)
Severe to very severe problem	444 (2.4)	298 (67.1)	146 (32.9)
Undetermined	3468 (18.7)	3079 (88.8)	389 (11.2)
Problems associated with hallucinations or delusions			
No problem	5619 (30.3)	4411 (78.5)	1208 (21.5)
Minor problem	1824 (9.8)	1132 (62.1)	692 (37.9)
Mild problem	2913 (15.7)	1454 (49.9)	1459 (50.1)
Moderately severe problem	3029 (16.3)	1361 (44.9)	1668 (55.1)
Severe to very severe problem	1722 (9.3)	693 (40.2)	1029 (59.8)
Undetermined	3462 (18.6)	3081 (89.0)	381 (11.0)
Problems with depressed mood			
No problem	4077 (22.0)	1653 (40.5)	2424 (59.5)
Minor problem	3267 (17.6)	1748 (53.5)	1519 (46.5)
Mild problem	3794 (20.4)	2561 (67.5)	1233 (32.5)
Moderately severe problem	2807 (15.1)	2184 (77.8)	623 (22.2)
Severe to very severe problem	1183 (6.4)	929 (78.5)	254 (21.5)
Undetermined	3441 (18.5)	3057 (88.8)	384 (11.2)
Problems with relationships			
No problem	4936 (26.6)	3087 (62.5)	1849 (37.5)
Minor problem	3590 (19.3)	2160 (60.2)	1430 (39.8)
Mild problem	3740 (20.1)	2207 (59.0)	1533 (41.0)
Moderately severe problem	2025 (10.9)	1155 (57.0)	870 (43.0)
Severe to very severe problem	655 (3.5)	376 (57.4)	279 (42.6)
Undetermined	3623 (19.5)	3147 (86.9)	476 (13.1)
Problems with activities of daily living			
No problem	6825 (36.8)	4292 (62.9)	2533 (37.1)
Minor problem	3369 (18.1)	1993 (59.2)	1376 (40.8)
Mild problem	2864 (15.4)	1647 (57.5)	1217 (42.5)
Moderately severe problem	1494 (8.1)	802 (53.7)	692 (46.4)
Severe to very severe problem	453 (2.4)	275 (60.7)	178 (39.3)
Undetermined	3564 (19.2)	3123 (87.6)	441 (12.4)
Problems with living conditions			
No problem	7989 (43.0)	4893 (61.3)	3096 (38.8)
Minor problem	2570 (13.8)	1534 (59.7)	1036 (40.3)
Mild problem	1773 (9.6)	1021 (57.6)	752 (42.4)
Moderately severe problem	1053 (5.7)	603 (57.3)	450 (42.7)
Severe to very severe problem	1135 (6.1)	685 (60.4)	450 (39.7)
Undetermined	4049 (21.8)	3396 (83.9)	653 (16.1)
Problems with occupation and activities			
No problem	5932 (32.0)	3681 (62.1)	2251 (38.0)
Minor problem	3315 (17.9)	1981 (59.8)	1134 (40.2)
Mild problem	3259 (17.6)	1894 (59.0)	1365 (41.9)
Moderately severe problem	1453 (7.8)	857 (59.0)	596 (41.0)
Severe to very severe problem	623 (3.4)	371 (59.6)	252 (40.5)
Undetermined	3987 (21.5)	3348 (84.0)	639 (16.0)

Note. Undetermined category represents missing data in the variable

^a^ Percentages may not add up to 100% due to rounding to one decimal place

### Ethnicity and involuntary hospitalisation

The ethnicities with the highest proportion of involuntary admission were Asian Chinese (52.3%), followed by Black African (51.7%), Black Caribbean (50.3%), and Black British or other Black background (48.8%). The lowest proportion of involuntary admissions was in the White British ethnicity (22.7%). In the unadjusted logistic regression model (Table 2), all minoritised ethnicities, except for White Irish and Mixed ethnicity White and Black African, were more likely to be involuntarily admitted than the White British group.

In the fully adjusted model (Table 2), increased odds of involuntary over voluntary hospitalisation were observed in the majority of ethnicities compared to the White British group. These were among service-users who are Asian Chinese (adjusted OR = 2.28, 95% CI: 1.53–3.41); Black African (aOR = 1.80, 95% CI: 1.57–2.07); Black British or any other Black background (aOR = 1.75, 95% CI: 1.55–1.98); Asian Bangladeshi (aOR = 1.68, 95% CI: 1.04–2.72); Asian Pakistani (aOR = 1.67, 95% CI: 1.16–2.41); Other ethnic background (aOR = 1.66, 95% CI: 1.41–1.96); Black Caribbean (aOR = 1.63, 95% CI: 1.40–1.89); Other White background (aOR = 1.49, 95% CI: 1.29–1.73); Asian British or any other Asian background (aOR = 1.47, 95% CI: 1.20–1.79); and Asian Indian (aOR = 1.43, 95% CI: 1.08–1.91).

No significant associations with involuntary hospitalisation were observed when comparing the White British group to Other Mixed ethnic background (aOR = 1.18, 95% CI: 0.82–1.71); Mixed ethnicity White and Black Caribbean (aOR = 1.08, 95% CI: 0.78–1.51); White Irish (aOR = 1.01, 95% CI: 0.79–1.28); and Mixed ethnicity White and Black African groups (aOR = 0.94, 95% CI: 0.55–1.60).

### Sociodemographic characteristics and involuntary hospitalisation

With regards to sociodemographic variables, the main findings were that:

- The only age groups with higher odds of involuntary admission were 18–24 and 50–64 years old, compared to the reference group of 35–49, the age group with the lowest proportion of involuntary admission.
- Women had higher odds of involuntary admission when compared to men, although the magnitude of the effect was very small.
- There were no significant associations for any deprivation quintiles with involuntary admission.
- Homeless service-users had a decreased odds of involuntary admission compared to those with a home. Instead, these individuals were more likely to be voluntarily admitted.
- Individuals classified as migrants had an increased odds of involuntary admission compared to people without this status.

**Table 2:** Multivariable logistic regression of unadjusted and fully adjusted associations with involuntary admission under MHA sects. 2,3,4, or 5(2) at first hospital admission

	OR (95% CI)		
Variable	Unadjusted	Fully adjusted	*p*-value
Ethnicity			
White British	Reference group		
White Irish	1.13 (0.92–1.39)	1.01 (0.79–1.28)	0.942
Other White background	2.28 (2.05–2.53)	1.49 (1.29–1.73)	< 0.001
Black African	3.64 (3.29–4.03)	1.80 (1.57–2.07)	< 0.001
Black Caribbean	3.44 (3.03–3.91)	1.63 (1.40–1.89)	< 0.001
Black British / Other Black background	3.24 (2.92–3.60)	1.75 (1.55–1.98)	< 0.001
Asian Bangladeshi	2.97 (1.96–4.51)	1.68 (1.04–2.72)	0.034
Asian Indian	1.93 (1.51–2.47)	1.43 (1.08–1.91)	0.013
Asian Pakistani	2.48 (1.80–3.41)	1.67 (1.16–2.41)	0.006
Asian Chinese	3.73 (2.63–5.28)	2.28 (1.53–3.41)	< 0.001
Asian British / Other Asian background	2.25 (1.91–2.65)	1.47 (1.20–1.79)	< 0.001
White and Black African	1.55 (0.97–2.47)	0.94 (0.55–1.60)	0.816
White and Black Caribbean	1.45 (1.09–1.94)	1.08 (0.78–1.51)	0.635
Other Mixed background	1.66 (1.20–2.29)	1.18 (0.82–1.71)	0.374
Other ethnic background	2.40 (2.10–2.73)	1.66 (1.41–1.96)	< 0.001
Age			
18–24	1.53 (1.40–1.68)	1.24 (1.11–1.38)	< 0.001
25–34	1.22 (1.13–1.33)	1.10 (1.00-1.21)	0.054
35–49	Reference group		
50–64	1.20 (1.10–1.32)	1.23 (1.10–1.38)	< 0.001
65–99	1.31 (1.18–1.46)	1.00 (0.87–1.15)	0.977
Gender			
Male	Reference group		
Female	1.04 (0.98–1.10)	1.11 (1.03–1.19)	0.007
Area-level deprivation			
1st quintile (least deprived)	Reference group		
2nd quintile	1.30 (1.16–1.45)	1.10 (0.97–1.25)	0.154
3rd quintile	1.31 (1.17–1.47)	1.08 (0.95–1.23)	0.260
4th quintile	1.37 (1.22–1.53)	1.09 (0.96–1.24)	0.177
5th quintile (most deprived)	1.34 (1.20–1.50)	1.08 (0.95–1.23)	0.242
Homeless			
No	Reference group		
Yes	0.37 (0.34–0.40)	0.62 (0.55–0.71)	< 0.001
Migrant status			
No	Reference group		
Yes	2.18 (2.02–2.35)	1.15 (1.02–1.29)	0.020

Note. Fully adjusted column includes adjustment for all variables in the table, as well as adjustment for psychiatric diagnosis and Health of the Nation Outcome Scales items (as seen in Table 1). *p-*value corresponds to fully adjusted analysis

### Interaction analysis

Contrast analyses and stratified analyses for the unadjusted models (Table 3 and Supplementary Material Tables S3-S7) suggested that the relationship between ethnicity and involuntary hospitalisation changed depending on four key sociodemographic variables. These were: (a) age [χ^2^ (*df =* 56, *N* = 18569) = 85.03, *p* = 0.007], (b) area-level deprivation [χ^2^ (70, 18569) = 103.39, *p* = 0.006], (c) housing situation/homelessness [χ^2^ (14, 18569) = 67.22, *p* < 0.001], and (d) migrant status [χ^2^ (28, 18569) = 48.19, *p* = 0.01]. In the analyses adjusted for all sociodemographic variables and the clinical factors of psychiatric diagnosis and HoNOS (Table 3), there was no longer an interaction between ethnicity and these sociodemographic variables in relation to involuntary hospitalisation in the contrast analyses. Due to the exploratory nature of the study, we present details of the interactions observed in crude models, due to their relevance to the use of the MHA, in line with other statistics [22].

**Table 3:** Interaction analysis of ethnicity and sociodemographic variables with involuntary admission as the outcome using contrast analysis to compare models with and without the interaction terms specified below

	Unadjusted model ^a^				Fully adjusted model ^b^			
Interaction terms	*N*	χ^2^	*df*	*p*-value	*N*	χ^2^	*df*	*p*-value
Ethnicity and Age	18,569	85.03	56	0.007	18,569	66.71	56	0.155
Ethnicity and Gender	18,596	20.76	14	0.108	18,596	14.29	14	0.428
Ethnicity and Area Deprivation	18,569	103.39	70	0.006	18,569	86.25	70	0.091
Ethnicity and Homelessness	18,569	67.22	14	< 0.001	18,569	15.55	14	0.342
Ethnicity and Migrant Status	18,569	48.19	28	0.010	18,569	22.42	28	0.762

*Note. N* = total sample. χ^2^ = chi-squared statistic value. *df = degrees of freedom*

^a^ Unadjusted model represents the unadjusted interactions of ethnicity with the key sociodemographic variable

^b^ Fully adjusted model represents the adjusted interaction of ethnicity with the key sociodemographic variable, adjusting for all other sociodemographic variables in the table as well as psychiatric diagnosis and Health of the Nation Outcome Scales items

In crude models, interaction analysis indicated that the relationship between ethnicity and involuntary admission varied by age group. Odds ratios for disparities in involuntary admission (comparing minoritised ethnic groups to White British) were strongest in the younger age groups (18–24 years old) for service-users of Other White, Black African, Black Caribbean, and Black British ethnicities (Supplementary Table S3).

- Other White background aged 65–99 [(OR_65-99_ = 1.47, 95% CI: 1.02–2.10) vs. (OR_18-24_ = 2.60, 95% CI: 1.97–3.24), interaction term *p* = 0.014].
- Black African aged 65–99 [(OR_65-99 =_ 1.84, 95% CI: 1.18–2.88) vs. (OR_18-24_ = 4.08, 95% CI: 3.19–5.21), interaction term *p* = 0.002]
- Black Caribbean aged 65–99 [(OR_65-99 =_ 2.00, 95% CI: 1.53–2.62) vs. (OR_18-24_ = 4.17, 95% CI: 2.85–6.11), interaction term *p* = 0.002]
- Black British aged 50–64 [(OR_50-64_ = 2.45, 95% CI: 1.84–3.26) vs. (OR_18-24_ = 3.93, 95% CI: 3.15–4.92), interaction term *p* = 0.011]

By contrast, for Asian British service-users, the odds ratio for the disparities in involuntary admission was higher in the middle-aged group (35–49 years old) [(OR_35 − 49_ = 3.19, 95% CI: 2.40–4.23) vs. (OR_18 − 24_ = 1.82, 95% CI: 1.23–2.70), interaction term *p* = 0.024].

In the unadjusted interaction analysis, the relationship between ethnicity and involuntary admission varied depending on the level of deprivation in the area where the person resided. For Black Caribbean service-users, disparities in involuntary admission (i.e. odds ratios comparing minoritised ethnic groups to White British people) were higher for those living in the most deprived areas [(OR_quintile 5_ = 4.14, 95% CI: 3.15–5.43) vs. (OR_quintile 1_ = 2.24, 95% CI: 1.43–3.51), interaction term *p* = 0.022] (Supplementary Table S5). However, among other ethnic groups the odds ratio for involuntary admission was lower for those living in more deprived areas. Namely:

- Black British service-users [(OR_quintile 4_ = 2.56, 95% CI: 2.05–3.21) vs. (OR_quintile 1_ = 4.18, 95% CI: 2.97–5.90), interaction term *p* = 0.019].
- Asian Pakistani service-users [(OR_quintile 4_ = 0.73, 95% CI: 0.24–2.17) vs. (OR_quintile 1_ = 3.02, 95% CI: 1.47–6.18), interaction term *p* = 0.033].
- White and Black Caribbean mixed-ethnicity service-users [(OR_quintile 4_ = 0.71, 95% CI: 0.34–1.48) vs. (OR_quintile 1_ = 3.66, 95% CI: 1.51–8.87) interaction term *p* = 0.005].

In the interaction analysis of crude models, we observed that the relationship between ethnicity and involuntary admission varied depending on homelessness status. Interestingly, the main effect of homelessness was protective against involuntary admission. However, for some minoritised ethnicities, disparities in involuntary admission (i.e. odds ratios comparing minoritised ethnic groups to White British) were higher in the homeless group than in the non-homeless group (Supplementary Table S6). Namely, this effect was observed among:

- Other White service-users [(OR_homeless_ = 4.57, 95% CI: 3.54–5.91) vs. (OR_not homeless_ = 2.03, 95% CI: 1.80–2.29), interaction term *p* < 0.001]
- Black African service-users [(OR_homeless_ = 7.34, 95% CI: 5.47–9.84) vs. (OR_not homeless_ = 3.08, 95% CI: 2.76–3.43), interaction term *p* < 0.001]
- Black Caribbean service-users [(OR_homeless_ = 4.96, 95% CI: 3.26–7.54) vs. (OR_not homeless_ = 2.98, 95% CI: 2.61–3.41), interaction term *p* = 0.024]
- Asian Chinese service-users [(OR_homeless_ = 13.05, 95% CI: 4.48–38.03) vs. (OR_not homeless_ = 2.93, 95% CI: 2.03–4.24), interaction term *p* = 0.01]
- Other ethnic background service-users [(OR_homeless_ = 5.16, 95% CI: 3.53–7.53) vs. (OR_not homeless_ = 2.01, 95% CI: 1.75–2.32), interaction term *p* < 0.001]

In the main analysis, migrants were at a higher risk for involuntary admission. However, in crude models, interaction analysis indicated that the relationship between ethnicity and involuntary admission varied depending on migrant status. The disparity in involuntary admission between people of Black African heritage and White British was higher (almost double the odds ratio) in the non-migrant group compared to the migrant group [(OR_non−migrant_ = 6.41, 95% CI: 4.48–9.16) vs. (OR_migrant_ = 3.38 95% CI: 2.27–5.03), interaction term *p* = 0.019] (Supplementary Table S7).

## Discussion

This retrospective cohort study examined the ethnic inequalities in voluntary and involuntary hospital admission in 18,569 service-users on their first admission to SLaM, adjusting for several sociodemographic and clinical factors. A further aim was to identify any intersectional effects of ethnicity with sociodemographic factors concerning involuntary admission. The study found evidence that compared to White British people there was a higher likelihood of involuntary hospitalisation in most minoritised ethnicities, but not in White Irish, Mixed ethnicity White and Black Caribbean, Mixed ethnicity White and Black African, and Other Mixed ethnic background. On analysis of interaction in the unadjusted models, there were interactions between ethnicity and age, deprivation, homelessness, and migrant status in relation to involuntary hospitalisation, but these differences were no longer significant in the fully adjusted models.

### Ethnicity

This study identified inequalities among minoritised ethnicities and involuntary hospitalisation that align with previous studies’ findings [8, 10, 11, 16, 32–34]. A possible explanation for this may be due to reduced access to resources driven by patient and service-level disadvantages, that affect ethnic minorities, leading to adverse pathways into mental health care and potentially higher levels of unmet care needs [9, 11, 35–38]. Interestingly, these associations do not hold for individuals of any Mixed ethnicity in the present study, suggesting these disadvantages in access to care may be minimised when one parent is of White British ethnicity. White Irish people also did not have an increased likelihood of involuntary admission.

The ethnic group with the strongest association with involuntary admission was the Asian Chinese group, with over twice the odds for involuntary admission compared to White British people. The magnitude of differences is similar to what was observed in a recent meta-analysis [11]. Black African service-users were almost twice as likely to be involuntarily admitted - a finding that is consistent across several studies [11, 13, 15]. The increased likelihood of involuntary admission among service-users of Black Caribbean and South Asian descent also aligns with previous studies [11].

### Sociodemographic variables

#### Age

The present study identified an association between age and involuntary hospitalisation, observed in the age groups of 18–24 and 50–64, as compared to people aged 35–49. The current study’s findings of the greatest risk of involuntary admission in the youngest age category of 18–24 are in keeping with that of Weich et al. [10] observed using national data. However, there is some divergence in previous literature that reports no associations [39–41], possibly due to these studies operationalising age as a continuous variable.

#### Gender

This study agrees with previous literature that reports an increased likelihood of involuntary hospitalisation among women [41, 42]. However, this association is not always seen across studies, as some have found evidence of increased risk in males, including in a recent literature review and meta-analyses [10, 21, 43–45]. Exploring the differences across literature suggests that these inconsistencies may be due to differences in analytical and recruitment methods, such as not adjusting for potential confounders [43] or restricting the cohort to one diagnostic group [45].

While the present study did not find interactions in either the unadjusted or fully adjusted models, findings in previous literature have suggested that gender plays a role in modifying risk for minoritised ethnic groups. For example a study by Mann et al. [12] observed, in stratified adjusted models, a greater likelihood of involuntary admission in women over men across many minoritised ethnicities when compared to White British people, although no interaction analyses were conducted. Similarly, a recent meta-analysis also observed that a higher proportion of women in a study was a predictor for involuntary hospitalisation among those who are Black Caribbean, ‘unspecified’ Black and South Asian people [11]. The higher likelihood for women may be related to unaddressed specific healthcare needs [45, 46], compounded disempowerment in negotiating mental healthcare [35, 48], and barriers to accessing services [49].

### Area-level deprivation

The findings for area-level deprivation differed from other studies. While this study did not find any associations between area-level deprivation and involuntary hospitalisation after adjusting for sociodemographic and clinical factors, other studies were able to find evidence of this [10, 50].

### Housing situation / homelessness

In this study, we observed that homeless service-users were less likely to be there involuntarily admitted compared to those who were not homeless. In the unadjusted interaction and stratified analyses, we observed that for some minoritised ethnicities, disparities in involuntary admission (i.e. odds ratios comparing minoritised ethnic groups to White British) were higher in the homeless group than in the non-homeless group. In the fully adjusted model, no significant interactions between ethnicity and homelessness were observed. The finding of the main association of reduced involuntary admission among those who are homeless was unexpected, as a previous meta-analysis had found that homelessness was not a predictor for involuntary admission [21]. The protective effect observed in the present study may be due to mental health services having a reduced threshold for requiring inpatient care for those without a home, as it can provide food and shelter to a population associated with a greater risk of premature mortality [51].

### Migrant status

Regarding migration, the findings from this study are in line with others, as migrants were at higher risk of involuntary hospitalisation than voluntary hospitalisation [11, 23–25]. This increased risk may be due to challenges migrants face, such as worsening social climates and acculturation barriers [52, 53], barriers to accessing care [49], and institutional racism due to their visible minority status [54, 55].

### Limitations and strengths

To accommodate for missing data, higher-level categories of migration status were created by amalgamating different data sources, and an undetermined category was used to maintain power in analysis. Additional sociodemographic variables such as marital and employment status could not be included in the analysis due to a large amount of missing data, potentially resulting in residual confounding. Although information on sociodemographic variables and involuntary hospitalisation were recorded simultaneously, it is unlikely that the type of hospitalisation has influenced the sociodemographic profile. In contrast, the clinical variables of HoNOS may suffer from imperfect adjustment as the item used may have been recorded within 28 days after admission if there was no assessment before admission. Thus, for some service-users, their admission status may influence the assessment of symptoms on HoNOS. Also, this cohort and the SLaM catchment area had a higher proportion of people in the lowest socioeconomic groups, which does not accurately represent the spread within England [26]. Further, this study divided IMD scores into quintiles, which may mask subtle associations, potentially resulting in loss of information via aggregation. Involuntary admission was directly compared to voluntary, and examining this outcome as dichotomous may ignore the realities of how voluntary admission can occur under coercion [56] and how some service-users admitted involuntarily may still possess some level of ‘voluntariness’ [40].

The study’s strengths included utilising a large, diverse, and representative cohort, owing to the near-monopoly of mental health care SLaM has within the catchment. In part due to the use of EHRs, this study was also able to investigate and adjust for several sociodemographic and clinical risk factors identified in previous literature. Further, the study did not restrict the cohort by psychiatric diagnosis, whereas many others had limited participants to those with a psychotic disorder. In addition, due to the large sample size of the cohort, ethnicity could be captured on a granular level, allowing for the expression of heterogeneity between ethnicities, as opposed to grouping ethnicities under larger, often dissimilar classifications.

### Implications

The findings of this study show that the majority of minoritised ethnicities have a greater likelihood of being admitted for inpatient care involuntarily, compared to White British service users. These disparities are not explained by other sociodemographic factors, psychiatric diagnoses, or the severity of clinical symptoms at the time of admission. This inequality ought to be the focus of policies and interventions designed to improve previous care experiences and other mechanisms contributing to inequalities in the likelihood of involuntary admission [8, 9, 11, 15, 21, 25–38, 48].

The absence of significant interactions between ethnicity and specific sociodemographic factors, when considering the impact of all sociodemographic and clinical factors, limits our ability to provide further recommendations for clinical practice. Although a few exploratory findings were observed in the unadjusted models, some of the findings may warrant further investigation The lack of observation of statistically significant findings when considering interactions between only two sociodemographic factors at a time might be a methodological limitation. Future work could employ latent class analyses (LCA), longitudinal study designs, and other methodologies that differently address the multidimensionality of intersectionality. A multifactorial approach like LCA may better capture the interaction of multiple intersecting underprivileged social identities [57]. Qualitative studies with service users and mental health professionals suggest higher risk perceptions for young black men, associated with higher involuntary admissions [58]. We recommend future studies to employ analytical approaches that capture the interaction of multiple factors.

## Conclusion

This study provides evidence of ethnic inequalities as after adjustment for various sociodemographic and clinical factors, evidence of an increased likelihood of involuntary hospitalisation, was found among 10 out of 14 minoritised ethnicities compared to white British people. The study also observed an increased likelihood of involuntary hospitalisation among service-users who were younger, migrants, or women– with the finding in the latter being unexpected based on previous literature. The findings also suggest that there was no evidence of major intersectional effects after controlling for sociodemographic and clinical factors.

## Electronic supplementary material

Below is the link to the electronic supplementary material.

Supplementary Material 1

## Data Availability

No datasets were generated or analysed during the current study.

## References

[1] Høyer G (2000) On the justification for civil commitment. Acta Psychiatrica Scand Supplement 101(399):65–71. 10.1111/j.0902-4441.2000.007s020[dash]16.x 10794032

[2] Välimäki M, Leino-Kilpi H, Helenius H (1996) Self-determination in clinical practice: the psychiatric patient’s point of view. Nurs Ethics 3(4):329–344. 10.1177/096973309600300406 8998035 10.1177/096973309600300406

[3] Department of Health and Social Care (2018) *Modernising the Mental Health Act: increasing choice, reducing compulsion*. Retrieved from https://assets.publishing.service.gov.uk/government/uploads/system/uploads/attachment_data/file/778897/Modernising_the_Mental_Health_Act_-_increasing_choice__reducing_compulsion.pdf

[4] Akther SF, Molyneaux E, Stuart R, Johnson S, Simpson A, Oram S (2019) Patients’ experiences of assessment and detention under mental health legislation: systematic review and qualitative meta-synthesis. BJPsych Open 5(3):1–10. 10.1192/bjo.2019.19 10.1192/bjo.2019.19 PMC6520528 31530313

[5] Kjellin L, Candefjord I-L, Machl M, Westrin C-G, Eriksson K, Ekblom B, stman O (1993) Coercion in psychiatric care: problems of medical ethics in a comprehensive empirical study. Behav Sci Law 11(3):323–334. 10.1002/bsl.2370110309

[6] McEvoy JP, Applebaum PS, Apperson LJ, Geller JL, Freter S (1989) Why must some schizophrenic patients be involuntarily committed? The role of insight. Compr Psychiatr 30(1):13–17. 10.1016/0010-440x(89)90113-2 10.1016/0010-440x(89)90113-2 2564330

[7] Kallert TW (2008) Coercion in psychiatry. Curr Opin Psychiatry 21(5):485–489. 10.1097/YCO.0b013e328305e49f 18650692 10.1097/YCO.0b013e328305e49f

[8] Bhui K, Stansfeld S, Hull S, Priebe S, Mole F, Feder G (2003) Ethnic variations in pathways to and use of specialist mental health services in the UK: systematic review. Br J Psychiatry 182(FEB):105–116. 10.1192/bjp.182.2.105 12562737 10.1192/bjp.182.2.105

[9] Halvorsrud K, Nazroo J, Otis M, Hajdukova B, E., Bhui K (2018) Ethnic inequalities and pathways to care in psychosis in England: A systematic review and meta-analysis. BMC Med 16(1):1–17. 10.1186/s12916-018-1201-9 10.1186/s12916-018-1201-9 PMC6290527 30537961

[10] Weich, S., McBride, O., Twigg, L., Duncan, C., Keown, P., Crepaz-Keay, D.,… Bhui,K. (2017). Variation in compulsory psychiatric inpatient admission in England: a cross-classified,multilevel analysis. *The Lancet Psychiatry*, *4*(8), 619–626. 10.1016/S2215-0366(17)30207-9 10.1016/S2215-0366(17)30207-9 28647537

[11] Barnett P, Mackay E, Matthews H, Gate R, Greenwood H, Ariyo K, Smith S (2019) Ethnic variations in compulsory detention under the mental health act: a systematic review and meta-analysis of international data. Lancet Psychiatry 6(4):305–317. 10.1016/S2215-0366(19)30027-6 30846354 10.1016/S2215-0366(19)30027-6 PMC6494977

[12] Mann, F., Fisher, H. L., Major, B., Lawrence, J., Tapfumaneyi, A., Joyce, J.,… Johnson,S. (2014). Ethnic variations in compulsory detention and hospital admission for psychosis across four UK Early Intervention Services. *BMC Psychiatry*, *14*(1), 1–12. 10.1186/s12888-014-0256-1 10.1186/s12888-014-0256-1 PMC4173060 25214411

[13] Morgan C, Mallett R, Hutchinson G, Bagalkote H, Morgan K, Fearon P, Leff J (2005) Pathways to care and ethnicity. I: sample characteristics and compulsory admission: report from the ÆSOP study. Br J Psychiatry 186:281–289. 10.1192/bjp.186.4.281 15802683 10.1192/bjp.186.4.281

[14] Bowers L, Jones J, Simpson A (2009) The demography of nurses and patients on acute psychiatric wards in England. J Clin Nurs 18(6):884–892. 10.1111/j.1365-2702.2008.02362.x 19239667 10.1111/j.1365-2702.2008.02362.x

[15] Lawlor C, Johnson S, Cole L, Howard LM (2012) Ethnic variations in pathways to acute care and compulsory detention for women experiencing a mental health crisis. Int J Soc Psychiatry 58(1):3–15. 10.1177/0020764010382369 21059630 10.1177/0020764010382369 PMC3257000

[16] Gajwani R, Parsons H, Birchwood M, Singh SP (2016) Ethnicity and detention: are black and minority ethnic (BME) groups disproportionately detained under the mental health act 2007? Soc Psychiatry Psychiatr Epidemiol 51(5):703–711. 10.1007/s00127-016-1181-z 26886264 10.1007/s00127-016-1181-z PMC4846695

[17] Singh SP, Burns T, Tyrer P, Islam Z, Parsons H, Crawford MJ (2014) Ethnicity as a predictor of detention under the mental health act. Psychol Med 44(5):997–1004. 10.1017/S003329171300086X 23795603 10.1017/S003329171300086X

[18] Rotenberg M, Tuck A, Ptashny R, McKenzie K (2017) The role of ethnicity in pathways to emergency psychiatric services for clients with psychosis. BMC Psychiatry 17(1):1–11. 10.1186/s12888-017-1285-3 28407748 10.1186/s12888-017-1285-3 PMC5390361

[19] Borschmann RD, Gillard S, Turner K, Lovell K, Goodrich-purnell N, Chambers M (2010) Demographic and referral patterns of people detained under sect. 136 of the mental health act (1983) in a South London mental health trust from 2005 to 2008. Med Sci Law 50(1):15–18 20349688 10.1258/msl.2009.009003

[20] Crenshaw K (1989) Demarginalizing the intersection of race and sex: A black feminist critique of antidiscrimination doctrine, feminist theory, and antiracist politics. Univ Chic Legal Forum 1(8):39–52. 10.4324/9780429499142-5

[21] Walker S, Mackay E, Barnett P, Sheridan Rains L, Dalton-Locke C, Trevillion K, Johnson S (2019) Clinical and social factors associated with involuntary psychiatric hospitalisation: a systematic review, meta-analysis, and narrative synthesis. Lancet Psychiatry 6:1039–1053. 10.1016/S2352-4642(21)00089-4 31777340 10.1016/S2215-0366(19)30406-7 PMC7029280

[22] NHS Digital (2021) NHS Digital. Mental Health Act Statistics, Annual Figures. Retrieved from https://files.digital.nhs.uk/ED/8F6815/ment-heal-act-stat-eng-2020-21-summ-rep.pdf

[23] Curley A, Agada E, Emechebe A, Anamdi C, Ng XT, Duffy R, Kelly BD (2016) Exploring and explaining involuntary care: the relationship between psychiatric admission status, gender and other demographic and clinical variables. Int J Law Psychiatry 47:53–59. 10.1016/j.ijlp.2016.02.034 27033975 10.1016/j.ijlp.2016.02.034

[24] Kelly BD, Emechebe A, Anamdi C, Duffy R, Murphy N, Rock C (2015) Custody, care and country of origin: demographic and diagnostic admission statistics at an inner-city adult psychiatry unit. Int J Law Psychiatry 38:1–7. 10.1016/j.ijlp.2015.01.001 25634112 10.1016/j.ijlp.2015.01.001

[25] Rodrigues, R., Macdougall, A. G., Zou, G., Lebenbaum, M., Kurdyak, P., Li, L.,…Anderson, K. K. (2019). Risk of involuntary admission among first-generation ethnic minority groups with early psychosis: A retrospective cohort study using health administrative data. *Epidemiology and Psychiatric Sciences*, *29*(59), 1–8. 10.1017/S2045796019000556 10.1017/S2045796019000556 PMC8061249 31610825

[26] Perera G, Broadbent M, Callard F, Chang CK, Downs J, Dutta R, Stewart R (2016) Cohort profile of the South London and Maudsley NHS foundation trust biomedical research centre (SLaM BRC) case register: current status and recent enhancement of an electronic mental health Record-derived data resource. BMJ Open 6(3):1–22. 10.1136/bmjopen-2015-008721 10.1136/bmjopen-2015-008721 PMC4785292 26932138

[27] Stewart, R., Soremekun, M., Perera, G., Broadbent, M., Callard, F., Denis, M.,…Lovestone, S. (2009). The South London and Maudsley NHS Foundation Trust Biomedical Research Centre (SLAM BRC) case register: development and descriptive data. 10.1186/1471-244X-9-51 10.1186/1471-244X-9-51 PMC2736946 19674459

[28] Fernandes AC, Cloete D, Broadbent MTM, Hayes RD, Chang CK, Jackson RG, Callard F (2013) Development and evaluation of a de-identification procedure for a case register sourced from mental health electronic records. BMC Med Inf Decis Mak 13(1):1. 10.1186/1472-6947-13-71 10.1186/1472-6947-13-71 PMC3751474 23842533

[29] Department of Communities and Local Government (2010) The English Indices of Deprivation 2010. Retrieved from https://assets.publishing.service.gov.uk/government/uploads/system/uploads/attachment_data/file/6320/1870718.pdf

[30] Wing, J., Beevor, A., Curtis, R. H., Park, S. B., Hadden, S., & Burns, A. (1998). Health of the Nation Outcome Scales (HoNOS). *British Journal of Psychiatry*, *172*, 11–18. 10.4324/9781003076391-177 9534825 10.1192/bjp.172.1.11

[31] StataCorp (2017) Stata statistical software: release 15. StataCorp LLC, TX

[32] Audini B, Lelliott P (2002) Age, gender and ethnicity of those detained under part II of the mental health act 1983. Br J Psychiatry 180:222–226. 10.1192/bjp.180.3.222 11872514 10.1192/bjp.180.3.222

[33] Singh SP, Croudace T, Beck A, Harrison G (1998) Perceived ethnicity and the risk of compulsory admission. Soc Psychiatry Psychiatr Epidemiol 33(1):39–44. 10.1007/s001270050020 9448444 10.1007/s001270050020

[34] Singh SP, Greenwood NAN, White S, Churchill R (2007) Ethnicity and the mental health act 1983. Br J Psychiatry 191:99–105 17666492 10.1192/bjp.bp.106.030346

[35] Lawrence V, McCombie C, Nikolakopoulos G, Morgan C (2021) Ethnicity and power in the mental health system: experiences of white British and black Caribbean people with psychosis. Epidemiol Psychiatric Sci 30(12):1–7. 10.1017/S2045796020001043 10.1017/S2045796020001043 PMC8057456 33543688

[36] Morris RM, Sellwood W, Edge D, Colling C, Stewart R, Cupitt C, Das-Munshi J (2020) Ethnicity and impact on the receipt of cognitive-behavioural therapy in people with psychosis or bipolar disorder: an english cohort study. BMJ Open 10(12). 10.1136/bmjopen-2019-034913 10.1136/bmjopen-2019-034913 PMC7745324 33323425

[37] Freitas, D. F. De, Kadra, G., Megan, S., Shetty, H., Broadbent, M., Patel, R.,…Khondoker, M. (2022). Ethnic inequalities in clozapine use among people with treatment– resistant schizophrenia: a retrospective cohort study using data from electronic clinical records. *Social Psychiatry and Psychiatric Epidemiology*, (0123456789). 10.1007/s00127-022-02257-3 10.1007/s00127-022-02257-3 PMC9246775 35246709

[38] Freitas DF, Walker S, Nyikavaranda P et al (2022) Ethnic inequalities in involuntary admission under the mental health act: an exploration of mediation effects of clinical care prior to the first admission. Br J Psychiatry 222:27–36. 10.1192/bjp.2022.141 10.1192/bjp.2022.141 PMC10250681 36281471

[39] Aguglia A, Moncalvo M, Solia F, Maina G, Aguglia A, Moncalvo M, Maina G (2016) Involuntary admissions in Italy: the impact of seasonality. Int J Psychiatry Clin Pract 20(4):232–238. 10.1080/13651501.2016.1214736 27551753 10.1080/13651501.2016.1214736

[40] Balducci PM, Bernardini F, Pauselli L, Tortorella A, Compton MT (2017) Correlates of involuntary admission: findings from an Italian inpatient psychiatric unit. Psychiatria Danubina 29(4):490–496. 10.24869/psyd.2017.490 29197207 10.24869/psyd.2017.490

[41] Gou, L., Zhou, J., Xiang, Y., Zhu, X., Correll, C. U., Ungvari, G. S.,… Wang, X. (2014). Frequency of Involuntary Admissions and Its Associations With Demographic and Clinical Characteristics in China. *Archives of Psychiatric Nursing*, *28*(4), 272–276. 10.1016/j.apnu.2014.04.002 10.1016/j.apnu.2014.04.002 25017561

[42] Opjordsmoen, S., Friis, S., Melle, I., Haahr, U., Johannessen, J. O., Larsen, T.K.,… McGlashan, T. H. (2010). A 2-year follow-up of involuntary admission’s influence upon adherence and outcome in first-episode psychosis. *Acta Psychiatrica Scandinavica*, *121*(5), 371–376. 10.1111/j.1600-0447.2009.01536.x 10.1111/j.1600-0447.2009.01536.x 20085554

[43] Ielmini M, Caselli I, Poloni N, Gasparini A (2018) Compulsory versus voluntary admission in psychiatry: an observational study. Minerva Psichiatrica 59(3):129–134. 10.23736/s0391-1772.18.01967-2

[44] Cougnard A, Kalmi E, Desage A, Misdrahi D, Abalan F, Brun-Rousseau H, Verdoux H (2004) Factors influencing compulsory admission in first-admitted subjects with psychosis. Soc Psychiatry Psychiatr Epidemiol 39(10):804–809. 10.1007/s00127-004-0826-5 15669661 10.1007/s00127-004-0826-5

[45] Canova Mosele PH, Figueira C, Antônio G, Bertuol Filho A, Ferreira de Lima JAR, Calegaro VC (2018) Involuntary psychiatric hospitalization and its relationship to psychopathology and aggression. Psychiatry Res 265(March):13–18. 10.1016/j.psychres.2018.04.031 29680512 10.1016/j.psychres.2018.04.031

[46] Jankovic J, Parsons J, Jovanović N, Berrisford G, Copello A, Fazil Q, Priebe S (2020) Differences in access and utilisation of mental health services in the perinatal period for women from ethnic minorities - A population-based study. BMC Med 18(1):1–12. 10.1186/S12916-020-01711-W/FIGURES/2 32912196 10.1186/s12916-020-01711-w PMC7488566

[47] Wellesley Wesley, E., Patel, I., Kadra-Scalzo, G., Pritchard, M., Shetty, H., Broadbent,M.,… de Freitas, D. F. (2021). Gender disparities in clozapine prescription in a cohort of treatment-resistant schizophrenia in the South London and Maudsley case register. *Schizophrenia Research*, *232*, 68–76. 10.1016/j.schres.2021.05.006 10.1016/j.schres.2021.05.006 34022618

[48] Tseris EJ, Hart B, E., Franks S (2022) My voice was discounted the whole way through: A gendered analysis of women’s experiences of involuntary mental health treatment. Affilia - Feminist Inq Social Work 37(4):645–663. 10.1177/08861099221108714

[49] Nyikavaranda P, Pantelic M, Jones CJ, Paudyal P, Tunks A, Llewellyn CD (2023) Barriers and facilitators to seeking and accessing mental health support in primary care and the community among female migrants in Europe: a feminisms systematic review. Int J Equity Health 22(1):1–21. 10.1186/s12939-023-01990-8 37752502 10.1186/s12939-023-01990-8 PMC10523615

[50] Keown P, Murphy H, McKenna D, McKinnon I (2018) Changes in the use of the mental health act 1983 in England 1984/85 to 2015/16. Br J Psychiatry 213(4):595–599. 10.1192/bjp.2018.123 30070183 10.1192/bjp.2018.123

[51] Yohanna D (2013) Deinstitutionalization of people with mental illness: causes and consequences. Am Med Association J Ethics 15(10):886–891 10.1001/virtualmentor.2013.15.10.mhst1-1310 24152782

[52] Frost DM (2020) Hostile and harmful: structural stigma and minority stress explain increased anxiety among migrants living in the united Kingdom after the brexit referendum. J Consult Clin Psychol 88(1):75–81. 10.1037/ccp0000458 31647276 10.1037/ccp0000458

[53] Choy B, Arunachalam K, G. S, Taylor M, Lee A (2021) Systematic review: acculturation strategies and their impact on the mental health of migrant populations. Public Health Pract 2. 10.1016/j.puhip.2020.100069 10.1016/j.puhip.2020.100069 PMC9461568 36101596

[54] Terhune J, Dykxhoorn J, Mackay E, Hollander AC, Kirkbride JB, Dalman C (2020) Migrant status and risk of compulsory admission at first diagnosis of psychotic disorder: A population-based cohort study in Sweden. Psychol Med 1–10. 10.1017/S0033291720002068 10.1017/S0033291720002068 PMC8842197 32578529

[55] McKenzie K, Bhui K (2007) Institutional racism in mental health care. BMJ 334(7595):645. 10.1136/bmj.39161.665579.BE 17395908 10.1136/bmj.39163.395972.80 PMC1839175

[56] Katsakou C, Marougka S, Garabette J, Rost F, Yeeles K, Priebe S (2011) Why do some voluntary patients feel coerced into hospitalisation? A mixed-methods study. Psychiatry Res 187(1–2):275–282. 10.1016/j.psychres.2011.01.001 21272940 10.1016/j.psychres.2011.01.001

[57] Goodwin L, Gazard B, Aschan L, MacCrimmon S, Hotopf M, Hatch SL (2018) Taking an intersectional approach to define latent classes of socioeconomic status, ethnicity and migration status for psychiatric epidemiological research. Epidemiol Psychiatric Sci 27(6):589–600. 10.1017/S2045796017000142 10.1017/S2045796017000142 PMC6998994 28390448

[58] Department of Health and Social Care (2019) *Focus groups: a qualitative exploration of perspectives on the Mental Health Act and people of African and Caribbean descent*. Retrieved from https://assets.publishing.service.gov.uk/media/5c6596e140f0b676df6e4746/Independent_Review_of_the_Mental_Health_Act_1983_-_supporting_documents.pdf

