# Supplementary material for: Ethnicity and involuntary hospitalisation: a study of intersectional effects
Source: Soc Psychiatry Psychiatr Epidemiol. 2025 Apr 16;60(9):2061–75. doi: 10.1007/s00127-025-02898-0 (PMC12378639; doi:10.1007/s00127-025-02898-0)
Supplement: Supplementary file 1 — Supplementary Material 1 [file 127_2025_2898_MOESM1_ESM.docx]

**Supplementary materials**

**Study:** Ethnicity and Involuntary Hospitalisation: A Study of Intersectional Effects

**Authors:** Rooble Ali, Susan Walker, Patrick Nyikavaranda, Johnny Downs, Rashmi Patel, Mizanur Khondoker, Kamaldeep Bhui, Richard D. Hayes, Daniela Fonseca de Freitas

Table S1. Sociodemographic characteristics stratified by ethnicity

| Sociodemographic Characteristics | White British  *n* (%) | White Irish  *n* (%) | Other White *n* (%) | Black African *n* (%) | Black Caribbean *n* (%) | Black British *n* (%) | Asian Bangladeshi *n* (%) | Asian Indian *n* (%) | Asian Pakistani *n* (%) | Asian Chinese *n* (%) | Asian British *n* (%) | WB African *n* (%) | WB Caribbean *n* (%) | Other Mixed *n* (%) | Other ethnicity *n* (%) |
| --- | --- | --- | --- | --- | --- | --- | --- | --- | --- | --- | --- | --- | --- | --- | --- |
| Age |  |  |  |  |  |  |  |  |  |  |  |  |  |  |  |
| 18-24 | 1018 (12.4) | 32 (6.2) | 285 (15.4) | 395 (19.5) | 125  (10.9) | 539 (28.6) | 15  (16.7) | 32  (11.2) | 42  (26.4) | 35  (26.9) | 126  (19.1) | 24  (28.9) | 61  (26.9) | 49  (28.7) | 228  (20.9) |
| 25-34 | 1677 (20.4) | 76 (14.8) | 604 (32.6) | 651 (32.1) | 187  (16.3) | 513  (27.2) | 39  (43.3) | 58 (20.2) | 43  (27.0) | 48  (36.9) | 186  (28.1) | 21  (25.3) | 66  (29.1) | 62  (36.3) | 340  (31.11) |
| 35-49 | 2579 (31.3) | 164 (32.0) | 625 (33.8) | 674 (33.2) | 324  (28.3) | 580  (30.7) | 25  (27.8) | 94  (32.8) | 47  (29.6) | 27 (20.8) | 222  (33.6) | 27  (32.5) | 67  (29.5) | 45  (26.3) | 335  (30.7) |
| 50-64 | 1591 (19.3) | 98 (19.1) | 203 (11.0) | 229 (11.3) | 255  (22.3) | 221  (11.7) | <10% | 57  (19.9) | 16  (10.1) | <10% | 85  (12.9) | <10% | 21  (9.3) | <10% | 145  (13.3) |
| 65-99 | 1375 (16.7) | 143 (27.9) | 135 (7.3) | 82  (4.0) | 254  (22.2) | 34  (1.8) | <5% | 46  (16.0) | <10% | <10% | 42  (6.4) | <5% | <10% | <5% | 45  (4.1) |
| Gender |  |  |  |  |  |  |  |  |  |  |  |  |  |  |  |
| Male | 4618  (56.0) | 310  (60.4) | 1052  (56.8) | 1054  (51.9) | 616  (53.8) | 1102  (58.4) | 48  (53.3) | 156  (54.4) | 74  (46.5) | 36  (27.7) | 399  (60.4) | 40  (48.2) | 111  (48.9) | 88  (51.5) | 639  (58.5) |
| Female | 3622  (44.0) | 203  (39.6) | 800 (43.2) | 977 (48.1) | 529  (46.2) | 785  (41.6) | 42  (46.7) | 131 (45.6) | 85  (53.5) | 94  (72.3) | 262  (39.6) | 43  (51.8) | 116  (51.1) | 83  (48.5) | 454  (41.5) |
| Area-level deprivation |  |  |  |  |  |  |  |  |  |  |  |  |  |  |  |
| 1^st^ quintile | 1487  (18.1) | 46  (9.0) | 183  (9.9) | 132  (6.5) | 87  (7.6) | 150  (8.0) | <10% | 63  (22.0) | 31  (19.5) | 19  (14.6) | 83  (12.6) | <5% | 20  (8.8) | 25  (14.6) | 102  (9.3) |
| 2^nd^ quintile | 1582 (19.2) | 86  (16.8) | 316  (17.1) | 315  (15.5) | 208  (18.2) | 317  (16.8) | 15  (16.7) | 54  (18.8) | 41  (25.8) | 24  (18.5) | 130  (19.7) | <10% | 48  (21.2) | 33  (19.3) | 191  (17.5) |
| 3^rd^ quintile | 1485 (18.0) | 89  (17.4) | 372  (20.1) | 413  (20.3) | 238  (20.8) | 386  (20.5) | <15% | 53  (18.5) | 25  (15.7) | 22  (16.9) | 122  (18.5) | 22  (26.5) | 51  (22.5) | 37  (21.6) | 240 (22.0) |
| 4^th^ quintile | 1499  (18.2) | 108  (21.1) | 321  (17.3) | 458  (22.6) | 248  (21.7) | 423  (22.4) | 21  (23.3) | 38  (13.2) | 21  (13.2) | 26  (20.0) | 139  (21.0) | 21  (25.3) | 48  (21.2) | 29  (17.0) | 207  (18.9) |
| 5^th^ quintile | 1362 (16.5) | 105  (20.5) | 254  (13.7) | 465  (22.9) | 269  (23.5) | 414  (21.9) | 30  (33.3) | 48  (16.7) | 30  (18.9) | 20  (15.4) | 115  (17.4) | 18  (21.7) | 39  (17.2) | 28  (16.4) | 190  (17.4) |
| Undetermined | 825 (10.0) | 79  (15.4) | 406  (21.9) | 248  (12.2) | 95  (8.3) | 197  (10.4) | <10% | 31  (10.8) | <10% | 19  (14.6) | 72  (10.9) | <15% | 21  (9.3) | 19  (11.1) | 163  (14.9) |
| Homeless |  |  |  |  |  |  |  |  |  |  |  |  |  |  |  |
| No | 6342 (77.0) | 362 (70.6) | 1428  (77.1) | 1779 (87.6) | 1032  (90.1) | 1626  (86.2) | 75  (83.3) | 239  (83.3) | 145  (91.2) | 116  (89.2) | 573  (86.7) | 69  (83.1) | 181  (79.7) | 140  (81.9) | 951  (87.0) |
| Yes | 1898 (23.0) | 151  (29.4) | 424 (22.9) | 252  (12.4) | 113  (9.9) | 261  (13.8) | 15  (16.7) | 48  (16.7) | <10% | <15% | 88  (13.3) | <20% | 46  (20.3) | 31  (18.1) | 142  (13.0) |
| Migrant status |  |  |  |  |  |  |  |  |  |  |  |  |  |  |  |
| No | 5476  (66.5) | 103 (20.1) | 113 (6.1) | 131 (6.4) | 234  (20.4) | 934 (49.5) | 19  (21.1) | 69 (24.0) | 36  (22.6) | <10% | 85  (12.9) | 32  (38.6) | 125  (55.1) | 68  (39.8) | 79  (7.2) |
| Yes | 149  (1.8) | 287  (56.0) | 1372 (74.1) | 1348 (66.4) | 369  (32.2) | 231 (12.2) | 46  (51.1) | 119 (41.5) | 77  (48.4) | 94  (72.3) | 391 (59.2) | 28  (33.7) | 18  (7.9) | 37 (21.6) | 533 (48.8) |
| Undetermined | 2615  (31.7) | 123 (24.0) | 367 (19.8) | 552 (27.2) | 542  (47.3) | 772 (38.3) | 25  (27.8) | 99 (34.5) | 46  (28.9) | 27 (20.8) | 185 (28.0) | 23  (27.7) | 84  (37.0) | 66 (38.6) | 481 (44.0) |

Note. Percentages may not add up to 100% due to rounding. Categories with less than 15 observations do not contain raw sample sizes. Undetermined category represents missing data in the variable. Black British category includes service users of Black British or Other Black backgrounds. Asian British category includes service users of Asian British or Other Asian backgrounds. WB African category stands for Mixed ethnicity White and Black African. WB Caribbean category stands for Mixed ethnicity White and Black Caribbean

Table S2. Multivariable logistic regression showing crude and adjusted associations with involuntary admission under MHA sections 2,3,4, or 5(2) at first admission

|  | OR (95% CI) | |  |
| --- | --- | --- | --- |
| Variable | Crude association | Fully adjusted | *p*-value |
| Ethnicity |  |  |  |
| White British | Reference group |  |  |
| White Irish | 1.13 (0.92-1.39) | 1.01 (0.79-1.28) | 0.942 |
| Other White background | 2.28 (2.05-2.53) | 1.49 (1.29-1.73) | <0.001 |
| Black African | 3.64 (3.29-4.03) | 1.80 (1.57-2.07) | <0.001 |
| Black Caribbean | 3.44 (3.03-3.91) | 1.63 (1.40-1.89) | <0.001 |
| Black British / Other Black background | 3.24 (2.92-3.60) | 1.75 (1.55-1.98) | <0.001 |
| Asian Bangladeshi | 2.97 (1.96-4.51) | 1.68 (1.04-2.72) | 0.034 |
| Asian Indian | 1.93 (1.51-2.47) | 1.43 (1.08-1.91) | 0.013 |
| Asian Pakistani | 2.48 (1.80-3.41) | 1.67 (1.16-2.41) | 0.006 |
| Asian Chinese | 3.73 (2.63-5.28) | 2.28 (1.53-3.41) | <0.001 |
| Asian British / Other Asian background | 2.25 (1.91-2.65) | 1.47 (1.20-1.79) | <0.001 |
| White and Black African | 1.55 (0.97-2.47) | 0.94 (0.55-1.60) | 0.816 |
| White and Black Caribbean | 1.45 (1.09-1.94) | 1.08 (0.78-1.51) | 0.635 |
| Other Mixed background | 1.66 (1.20-2.29) | 1.18 (0.82-1.71) | 0.374 |
| Other ethnic background | 2.40 (2.10-2.73) | 1.66 (1.41-1.96) | <0.001 |
| Sociodemographic factors |  |  |  |
| Age |  |  |  |
| 18-24 | 1.53 (1.40-1.68) | 1.24 (1.11-1.38) | <0.001 |
| 25-34 | 1.22 (1.13-1.33) | 1.10 (1.00-1.21) | 0.054 |
| 35-49 | Reference group |  |  |
| 50-64 | 1.20 (1.10-1.32) | 1.23 (1.10-1.38) | <0.001 |
| 65-99 | 1.31 (1.18-1.46) | 1.00 (0.87-1.15) | 0.977 |
| Gender |  |  |  |
| Male | Reference group |  |  |
| Female | 1.04 (0.98-1.10) | 1.11 (1.03-1.19) | 0.007 |
| Area-level deprivation |  |  |  |
| 1^st^ quintile (least deprived) | Reference group |  |  |
| 2^nd^ quintile | 1.30 (1.16-1.45) | 1.10 (0.97-1.25) | 0.154 |
| 3^rd^ quintile | 1.31 (1.17-1.47) | 1.08 (0.95-1.23) | 0.260 |
| 4^th^ quintile | 1.37 (1.22-1.53) | 1.09 (0.96-1.24) | 0.177 |
| 5^th^ quintile (most deprived) | 1.34 (1.20-1.50) | 1.08 (0.95-1.23) | 0.242 |
| Undetermined | 1.23 (1.08-1.39) | 1.23 (1.05-1.44) | 0.009 |
| Homeless |  |  |  |
| No | Reference group |  |  |
| Yes | 0.37 (0.34-0.40) | 0.62 (0.55-0.71) | <0.001 |
| Migrant status |  |  |  |
| No | Reference group |  |  |
| Yes | 2.18 (2.02-2.35) | 1.15 (1.02-1.29) | 0.020 |
| Undetermined | 1.97 (1.83-2.12) | 1.16 (1.06-1.27) | 0.002 |
|  |  |  |  |
| Psychiatric diagnosis (ICD-10)  Mental disorders due to known physiological conditions |  |  |  |
| No | Reference group |  |  |
| Yes | 1.09 (0.95-1.26) | 0.76 (0.64-0.91) | 0.002 |
| Mental and behavioural disorders due to psychoactive substance use |  |  |  |
| No | Reference group |  |  |
| Yes | 0.29 (0.26-0.32) | 0.56 (0.49-0.64) | <0.001 |
| Schizophrenia, schizotypal, delusional, and other non-mood psychotic disorders |  |  |  |
| No | Reference group |  |  |
| Yes | 3.55 (3.30-3.83) | 1.52 (1.39-1.66) | <0.001 |
| Affective psychosis |  |  |  |
| No | Reference group |  |  |
| Yes | 2.01 (1.74-2.32) | 1.63 (1.38-1.92) | <0.001 |
| Mood disorder |  |  |  |
| No | Reference group |  |  |
| Yes | 0.48 (0.44-0.53) | 0.67 (0.60-0.75) | <0.001 |
| Anxiety, dissociative, stress-related, somatoform, and other nonpsychotic disorders |  |  |  |
| No | Reference group |  |  |
| Yes | 0.42 (0.36-0.47) | 0.66 (0.57-0.77) | <0.001 |
| Behavioural syndromes associated with physiological disturbances and physical factors |  |  |  |
| No | Reference group |  |  |
| Yes | 0.42 (0.31-0.58) | 0.47 (0.33-0.67) | <0.001 |
| Disorders of adult personality and behaviour |  |  |  |
| No | Reference group |  |  |
| Yes | 0.43 (0.36-0.52) | 0.63 (0.51-0.78) | <0.001 |
| Intellectual disabilities |  |  |  |
| No | Reference group |  |  |
| Yes | 1.17 (0.79-1.71) | 0.96 (0.62-1.47) | 0.835 |
| Pervasive and specific developmental disorders |  |  |  |
| No | Reference group |  |  |
| Yes | 1.24 (0.78-1.99) | 0.98 (0.58-1.66) | 0.951 |
| Behavioural and emotional disorders with onset usually occurring in childhood and adolescence |  |  |  |
| No | Reference group |  |  |
| Yes | 0.86 (0.42-1.76) | 0.82 (0.37-1.82) | 0.626 |
| Health of the Nation Outcome Scale (HoNOS) |  |  |  |
| Overactive, aggressive, disruptive, or agitated behaviour |  |  |  |
| No problem | Reference group |  |  |
| Minor problem | 1.87 (1.71-2.05) | 1.53 (1.39-1.70) | <0.001 |
| Mild problem | 2.51 (2.29-2.75) | 1.84 (1.66-2.05) | <0.001 |
| Moderately severe problem | 3.69 (3.32-4.10) | 2.54 (2.24-2.87) | <0.001 |
| Severe to very severe problem | 5.01 (4.37-5.74) | 3.29 (2.80-3.87) | <0.001 |
| Undetermined | 0.31 (0.27-0.35) | 0.07 (0.03-0.19) | <0.001 |
| Non-accidental self-injury |  |  |  |
| No problem | Reference group |  |  |
| Minor problem | 0.61 (0.55-0.68) | 0.80 (0.71-0.90) | <0.001 |
| Mild problem | 0.38 (0.33-0.42) | 0.60 (0.52-0.69) | <0.001 |
| Moderately severe problem | 0.27 (0.24-0.31) | 0.57 (0.49-0.67) | <0.001 |
| Severe to very severe problem | 0.24 (0.20-0.28) | 0.57 (0.47-0.69) | <0.001 |
| Undetermined | 0.13 (0.11-0.14) | 1.35 (0.63-2.89) | 0.440 |
| Problem drinking or drug taking |  |  |  |
| No problem | Reference group |  |  |
| Minor problem | 1.07 (0.95-1.20) | 1.22 (1.07-1.40) | 0.003 |
| Mild problem | 1.02 (0.92-1.13) | 1.15 (1.02-1.31) | 0.028 |
| Moderately severe problem | 0.91 (0.82-1.02) | 1.24 (1.08-1.42) | 0.002 |
| Severe to very severe problem | 0.78 (0.68-0.90) | 1.18 (0.98-1.41) | 0.077 |
| Undetermined | 0.24 (0.22-0.27) | 1.65 (1.22-2.22) | 0.001 |
| Cognitive problems |  |  |  |
| No problem | Reference group |  |  |
| Minor problem | 1.51 (1.38-1.64) | 1.23 (1.11-1.36) | <0.001 |
| Mild problem | 1.73 (1.57-1.91) | 1.17 (1.05-1.32) | 0.007 |
| Moderately severe problem | 1.71 (1.51-1.93) | 1.00 (0.86-1.16) | 0.984 |
| Severe to very severe problem | 1.78 (1.46-2.16) | 0.99 (0.79-1.26) | 0.964 |
| Undetermined | 0.23 (0.20-0.26) | 0.90 (0.54-1.50) | 0.693 |
| Physical illness or disability problems |  |  |  |
| No problem | Reference group |  |  |
| Minor problem | 0.88 (0.80-0.97) | 0.93 (0.83-1.04) | 0.188 |
| Mild problem | 0.80 (0.72-0.88) | 0.84 (0.75-0.95) | 0.006 |
| Moderately severe problem | 0.69 (0.61-0.79) | 0.76 (0.65-0.88) | <0.001 |
| Severe to very severe problem | 0.67 (0.55-0.82) | 0.81 (0.64-1.02) | 0.074 |
| Undetermined | 0.17 (0.15-0.19) | 1.04 (0.64-1.69) | 0.863 |
| Problems associated with hallucinations or delusions |  |  |  |
| No problem | Reference group |  |  |
| Minor problem | 2.23 (1.99-2.50) | 1.39 (1.23-1.58) | <0.001 |
| Mild problem | 3.66 (3.33-4.04) | 1.78 (1.60-1.99) | <0.001 |
| Moderately severe problem | 4.48 (4.07-4.93) | 1.76 (1.58-1.97) | <0.001 |
| Severe to very severe problem | 5.42 (4.83-6.09) | 1.96 (1.72-2.25) | <0.001 |
| Undetermined | 0.45 (0.40-0.51) | 1.69 (1.05-2.74) | 0.032 |
| Problems with depressed mood |  |  |  |
| No problem | Reference group |  |  |
| Minor problem | 0.59 (0.54-0.65) | 0.68 (0.62-0.76) | <0.001 |
| Mild problem | 0.33 (0.30-0.36) | 0.50 (0.45-0.55) | <0.001 |
| Moderately severe problem | 0.19 (0.17-0.22) | 0.39 (0.34-0.44) | <0.001 |
| Severe to very severe problem | 0.19 (0.16-0.22) | 0.43 (0.36-0.52) | <0.001 |
| Undetermined | 0.09 (0.08-0.10) | 0.98 (0.56-1.71) | 0.947 |
| Problems with relationships |  |  |  |
| No problem | Reference group |  |  |
| Minor problem | 1.11 (1.01-1.21) | 1.09 (0.99-1.21) | 0.093 |
| Mild problem | 1.16 (1.06-1.27) | 1.11 (1.00-1.24) | 0.042 |
| Moderately severe problem | 1.26 (1.13-1.40) | 1.25 (1.10-1.42) | 0.001 |
| Severe to very severe problem | 1.24 (1.05-1.46) | 1.30 (1.06-1.59) | 0.012 |
| Undetermined | 0.25 (0.23-0.28) | 1.22 (0.88-1.68) | 0.232 |
| Problems with activities of daily living |  |  |  |
| No problem | Reference group |  |  |
| Minor problem | 1.17 (1.08-1.27) | 1.06 (0.96-1.18) | 0.262 |
| Mild problem | 1.25 (1.15-1.37) | 1.03 (0.92-1.16) | 0.582 |
| Moderately severe problem | 1.46 (1.31-1.64) | 1.22 (1.05-1.42) | 0.008 |
| Severe to very severe problem | 1.10 (0.90-1.33) | 0.85 (0.66-1.08) | 0.189 |
| Undetermined | 0.24 (0.21-0.27) | 1.24 (0.86-1.79) | 0.257 |
| Problems with living conditions |  |  |  |
| No problem | Reference group |  |  |
| Minor problem | 1.07 (0.97-1.17) | 0.99 (0.89-1.10) | 0.854 |
| Mild problem | 1.16 (1.05-1.29) | 1.05 (0.93-1.19) | 0.421 |
| Moderately severe problem | 1.18 (1.04-1.34) | 1.03 (0.88-1.20) | 0.742 |
| Severe to very severe problem | 1.04 (0.91-1.18) | 0.96 (0.82-1.13) | 0.603 |
| Undetermined | 0.30 (0.28-0.33) | 0.95 (0.76-1.18) | 0.627 |
| Problems with occupation and activities |  |  |  |
| No problem | Reference group |  |  |
| Minor problem | 1.10 (1.01-1.20) | 0.99 (0.89-1.10) | 0.880 |
| Mild problem | 1.18 (1.08-1.29) | 1.06 (0.95-1.19) | 0.265 |
| Moderately severe problem | 1.14 (1.01-1.28) | 0.96 (0.82-1.11) | 0.545 |
| Severe to very severe problem | 1.11 (0.94-1.31) | 0.99 (0.80-1.22) | 0.945 |
| Undetermined | 0.31 (0.28-0.34) | 1.12 (0.88-1.41) | 0.358 |

*Note.* Fully adjusted column includes adjustment for all variables in the table. *p­-*value corresponds to fully adjusted analysis.

Table S3. Stratified analysis (crude and fully adjusted) of the relationship between ethnicity involuntary admission under the MHA by age

| Ethnicity | 18-24 | | 25-34 | | 35-49 | | 50-64 | | 65-99 | |
| --- | --- | --- | --- | --- | --- | --- | --- | --- | --- | --- |
|  | Crude OR (95% CI) | FA OR ^a^ (95% CI) | Crude OR (95% CI) | FA OR ^a^ (95% CI) | Crude OR (95% CI) | FA OR ^a^ (95% CI) | Crude OR (95% CI) | FA OR ^a^ (95% CI) | Crude OR  (95% CI) | FA OR ^a^ (95% CI) |
| White British | (ref) | (ref) | (ref) | (ref) | (ref) | (ref) | (ref) | (ref) | (ref) | (ref) |
| White Irish | 1.49  (0.70-3.19) | 2.01  (0.82-4.93) | 1.35  (0.79-2.33) | 1.25  (0.66-2.38) | 0.83  (0.54-1.28) | 0.61  (0.37-1.00) | 1.00  (0.62-1.59) | 1.38  (0.78-2.46) | 1.11  (0.77-1.60) | 1.06  (0.67-1.65) |
| Other White background | 2.60  (1.97-3.42) | 1.67  (1.16-2.43) | 3.32  (2.71-4.06) | 1.92  (1.44-2.56) | 2.45  (2.02-2.97) | 1.28  (0.97-1.67) | 1.82  (1.34-2.46) | 1.46  (0.97-2.19) | 1.47  (1.02-2.10) | 1.09  (0.68-1.74) |
| Black African | 4.08  (3.19-5.21) | 1.98  (1.42-2.76) | 4.71  (3.87-5.73) | 2.35  (1.79-3.09) | 4.04  (3.37-4.84) | 1.50  (1.15- 1.94) | 4.03  (3.03-5.36) | 1.78  (1.23-2.60) | 1.84  (1.18-2.88) | 0.99  (0.56-1.73) |
| Black Caribbean | 4.17  (2.85-6.11) | 2.32  (1.47-3.66) | 4.31  (3.16-5.89) | 1.87  (1.29-2.70) | 3.95  (3.11-5.02) | 1.60  (1.20-2.14) | 3.38  (2.58-4.43) | 1.82  (1.30-2.56) | 2.00  (1.53-2.62) | 1.10  (0.77-1.57) |
| Black British | 3.93  (3.15-4.92) | 2.26  (1.73-2.95) | 4.27  (3.46-5.28) | 2.20  (1.71-2.82) | 3.42  (2.82-4.14) | 1.49  (1.17-1.87) | 2.45  (1.84-3.26) | 1.40  (0.99-2.00) | 2.70  (1.36-5.37) | 1.81  (0.83-3.95) |
| Asian Bangladeshi | 6.55  (2.22-19.36) | 5.47  (1.53-19.53) | 2.72  (1.41-5.25) | 1.33  (0.62-2.85) | 2.49  (1.09-5.66) | 1.32  (0.52-3.43) | 7.27  (1.41-37.63) | 3.31  (0.47-20.70) | 6.40  (0.66-61.67) | 4.64  (0.43-50.59) |
| Asian Indian | 1.97  (0.95-4.08) | 1.33  (0.58-3.07) | 2.86  (1.67-4.92) | 2.04  (1.08-3.86) | 2.28  (1.47-3.53) | 1.90  (1.12-3.22) | 1.14  (0.63-2.05) | 0.73  (0.36-1.48) | 1.79  (0.99-3.23) | 1.71  (0.85-3.46) |
| Asian Pakistani | 2.46  (1.31-4.61) | 2.17  (1.02-4.61) | 2.10  (1.10-4.03) | 1.39  (0.67-2.87) | 4.23  (2.37-7.57) | 1.99  (1.00-3.94) | 2.26  (0.84-6.11) | 2.11  (0.64-6.92) | 1.78  (0.54-5.85) | 1.40  (0.38-5.19) |
| Asian Chinese | 3.10  (1.57-6.10) | 2.54  (1.13-5.68) | 4.36  (2.44-7.78) | 2.33  (1.19-4.54) | 4.76  (2.22-10.19) | 1.97  (0.84-4.62) | 5.09  (1.48-17.48) | 6.90  (1.47-32.41) | 4.26  (1.06-17.13) | 5.90  (1.12-31.00) |
| Asian British | 1.82  (1.23-2.70) | 1.18  (0.58-3.07) | 2.40  (1.73-3.32) | 1.69  (1.14-2.50) | 3.19  (2.40-4.23) | 1.36  (0.95-1.94) | 2.35  (1.51-3.66) | 1.88  (1.08-3.26) | 2.13  (1.15-3.95) | 1.51  (0.74-3.08) |
| Mixed WB  African | 2.34  (1.03-5.34) | 0.90  (0.34-2.38) | 1.74  (0.67-4.53) | 1.14  (0.40-3.26) | 1.26  (0.51-3.14) | 0.64  (0.22-1.84) | 1.75  (0.42-7.34) | 1.28  (0.23-6.97) | 1.07  (0.10-11.79) | 4.45  (0.36-55.20) |
| Mixed WB Caribbean | 1.27  (0.71-2.26) | 1.18  (0.61-2.28) | 2.33  (1.38-3.92) | 1.46  (0.79-2.68) | 1.50  (0.86-2.63) | 1.06  (0.54-2.10) | 0.91  (0.33-2.50) | 0.46  (0.14-1.46) | 2.13  (0.68-6.65) | 1.41  (0.40-4.94) |
| Other Mixed background | 1.90  (1.05-3.46) | 1.61  (0.81-3.18) | 1.52  (0.85-2.71) | 0.87  (0.45-1.68) | 2.44  (1.31-4.52) | 1.20  (0.57-2.54) | 2.42  (0.74-7.99) | 1.99  (0.44-8.99) | 0.71  (0.07-6.85) | 0.76  (0.07-7.85) |
| Other ethnic background | 3.06  (2.27-4.11) | 2.18  (1.49-3.17) | 3.01  (2.35-3.86) | 1.95  (1.42-2.66) | 2.60  (2.04-3.31) | 1.59  (1.17-2.16) | 2.00  (1.41-2.83) | 1.51  (0.99-2.33) | 1.71  (0.94-3.10) | 1.07  (0.54-2.16) |

*Note*. *OR* odds ratio, *CI* confidence interval, *FA* fully adjusted, *WB* White and Black*.* ^a^ Fully adjusted model adjusts for all sociodemographic and clinical variables in the study. Broad confidence intervals suggest reduced power, due to small observations in the cell.

Table S4. Stratified analysis (crude and fully adjusted) of the relationship between ethnicity and involuntary admission under the MHA by gender

| Ethnicity | Male | | Female | |
| --- | --- | --- | --- | --- |
|  | Crude OR (95% CI) | FA OR^a^ (95% CI) | Crude OR (95% CI) | FA OR^a^ (95% CI) |
| White British | (ref) | (ref) | (ref) | (ref) |
| White Irish | 1.04  (0.79-1.37) | 0.84  (0.61-1.16) | 1.28  (0.93-1.75) | 1.32  (0.92-1.90) |
| Other White background | 2.22  (1.92-2.56) | 1.32  (1.08-1.60) | 2.36  (2.01-2.77) | 1.74  (1.40-2.16) |
| Black African | 3.60  (3.13-4.13) | 1.55  (1.28-1.88) | 3.67  (3.16-4.25) | 2.10  (1.71-2.58) |
| Black Caribbean | 3.71  (3.12-4.41) | 1.63  (1.33-2.00) | 3.14  (2.61-3.79) | 1.67  (1.34-2.09) |
| Black British | 3.63  (3.17-4.16) | 1.68  (1.43-1.99) | 2.78  (2.36-3.26) | 1.86  (1.53-2.26) |
| Asian Bangladeshi | 3.22  (1.82-5.70) | 1.65  (0.84-3.24) | 2.71  (1.47-4.99) | 1.77  (0.88-3.56) |
| Asian Indian | 1.85  (1.32-2.60) | 1.23  (0.83-1.82) | 2.02  (1.41-2.90) | 1.72  (1.13-2.62) |
| Asian Pakistani | 2.52  (1.58-4.03) | 1.59  (0.93-2.72) | 2.41  (1.55-3.73) | 1.86  (1.12-3.08) |
| Asian Chinese | 7.00  (3.49-14.05) | 5.10  (2.31-11.28) | 2.88  (1.91-4.35) | 1.99  (1.23-3.22) |
| Asian British | 2.15  (1.74-2.67) | 1.30  (1.00-1.68) | 2.41  (1.86-3.11) | 1.71  (1.25-2.30) |
| Mixed WB African | 1.69  (0.87-3.28) | 0.87  (0.41-1.86) | 1.42  (0.74-2.73) | 0.97  (0.45-2.10) |
| Mixed WB Caribbean | 1.75  (1.17-2.61) | 1.04  (0.66-1.67) | 1.19  (0.79-1.82) | 1.14  (0.70-1.86) |
| Other Mixed background | 2.20  (1.43-3.40) | 1.14  (0.69-1.88) | 1.18  (0.72-1.94) | 1.20  (0.69-2.12) |
| Other ethnic background | 2.45  (2.06-2.91) | 1.57  (0.69-1.88) | 2.34  (1.91-2.86) | 1.75  (1.36-2.25) |

*Note*. *OR* odds ratio, *CI* confidence interval, *FA* fully adjusted, *WB* White and Black*.* ^a^ Fully adjusted model adjusts for all sociodemographic and clinical variables in the study. Broad confidence intervals suggest reduced power, due to small observations in the cell.

Table S5. Stratified analysis (crude and fully adjusted) of the relationship between ethnicity and involuntary admission under the MHA, by area-level deprivation

| Ethnicity | Least deprived | | 2^nd^ quintile | | 3^rd^ quintile | | 4^th^ quintile | | Most deprived | |
| --- | --- | --- | --- | --- | --- | --- | --- | --- | --- | --- |
|  | Crude OR (95% CI) | FA OR^a^ (95% CI) | Crude OR (95% CI) | FA OR^a^ (95% CI) | Crude OR (95% CI) | FA OR^a^ (95% CI) | Crude OR (95% CI) | FA OR^a^ (95% CI) | Crude OR (95% CI) | FA OR^a^ (95% CI) |
| White British | (ref) | (ref) | (ref) | (ref) | (ref) | (ref) | (ref) | (ref) | (ref) | (ref) |
| White Irish | 1.44  (0.75-2.77) | 1.37  (0.64-2.92) | 0.94  (0.56-1.57) | 0.92  (0.50-1.69) | 1.36  (0.85-2.18) | 1.33  (0.77-2.31) | 0.88  (0.55-1.41) | 0.95  (0.54-1.67) | 1.13  (0.70-1.81) | 1.13  (0.64-1.99) |
| Other White background | 2.37  (1.72-3.28) | 1.42  (0.91-2.23) | 2.01  (1.56-2.59) | 1.24  (0.87-1.75) | 2.06  (1.62-2.62) | 1.41  (1.01-1.97) | 1.89  (1.47-2.44) | 1.48  (1.04-2.09) | 2.69  (2.03-3.56) | 1.79  (1.22-2.61) |
| Black African | 4.13  (2.87-5.95) | 2.27  (1.39-3.72) | 3.89  (3.03-4.99) | 1.79  (1.27-2.50) | 2.94  (2.35-3.70) | 1.29  (0.94-1.76) | 3.67  (2.95-4.57) | 1.95  (1.42-2.68) | 4.28  (3.42-5.36) | 1.94  (1.41-2.67) |
| Black Caribbean | 2.24  (1.43-3.51) | 1.13  (0.66-1.95) | 3.49  (2.60-4.69) | 1.60  (1.12-2.29) | 3.08  (2.32-4.08) | 1.57  (1.12-2.20) | 3.75  (2.84-4.94) | 1.82  (1.30-2.55) | 4.14  (3.15-5.43) | 1.96  (1.41-2.72) |
| Black British | 4.18  (2.97-5.90) | 2.61  (1.72-3.96) | 2.79  (2.18-3.58) | 1.47  (1.09-2.00) | 3.73  (2.95-4.71) | 2.02  (1.52-2.69) | 2.56  (2.05-3.21) | 1.29  (0.97-1.70) | 3.85  (3.05-4.86) | 1.99  (1.50-2.65) |
| Asian Bangladeshi | 18.31  (2.13-157.26) | 8.17  (0.65-102.87) | 1.13  (0.36-3.57) | 1.23  (0.33-4.61) | 14.81  (3.19-68.88) | 3.63  (0.71-18.47) | 2.31  (0.97-5.53) | 1.80  (0.65-5.03) | 2.54  (1.21-5.34) | 1.34  (0.57-3.16) |
| Asian Indian | 1.70  (0.99-2.94) | 1.43  (0.75-2.75) | 3.11  (1.39-6.98) | 2.40  (1.25-4.63) | 1.30  (0.71-2.39) | 0.99  (0.49-2.00) | 1.80  (0.92-3.51) | 1.08  (0.48-2.44) | 2.50  (1.38-4.52) | 2.04  (0.99-4.17) |
| Asian Pakistani | 3.02  (1.47-6.18) | 2.17  (0.94-5.02) | 3.26  (1.75-6.09) | 2.27  (1.09-4.70) | 3.04  (1.37-6.72) | 2.37  (0.97-5.82) | 0.73  (0.24-2.17) | 0.37  (0.11-1.25) | 3.34  (1.61-6.92) | 1.66  (0.70-3.97) |
| Asian Chinese | 5.03  (2.01-12.62) | 2.37  (0.77-7.29) | 3.11  (1.39-6.98) | 1.16  (0.46-2.93) | 2.74  (1.18-6.40) | 1.80  (0.63-5.16) | 2.26  (1.03-4.97) | 1.91 (0.75-4.86) | 3.81  (1.57-9.25) | 2.02 (0.77-5.32) |
| Asian British | 2.30  (1.45-3.64) | 1.54  (0.86-2.73) | 1.38  (0.94-2.04) | 0.69  (0.43-1.10) | 2.53  (1.73-3.69) | 1.77  (1.13-2.79) | 2.63  (1.85-3.75) | 2.08 (1.32-3.28) | 2.83  (1.91-4.19) | 1.77 (1.10-2.84) |
| Mixed WB African | 1.83  (0.17-20.25) | 1.35  (0.08-24.01) | 7.77  (1.50-40.22) | 3.60  (0.61-21.28) | 0.73  (0.25-2.18) | 0.36  (0.10-1.27) | 1.54  (0.62-3.85) | 0.85 (0.28-2.53) | 2.43  (0.93-6.32) | 1.47 (0.50-4.36) |
| Mixed WB Caribbean | 3.66  (1.51-8.87) | 2.21  (0.71-6.96) | 1.41  (0.76-2.63) | 1.23  (0.60-2.54) | 1.50  (0.82-2.75) | 1.38  (0.68-2.78) | 0.71  (0.34-1.48) | 0.50 (0.21-1.14) | 1.69  (0.85-3.39) | 1.53  (0.68-3.42) |
| Other Mixed background | 1.16  (0.46-2.92) | 0.71  (0.24-2.12) | 1.78  (0.87-3.64) | 1.25  (0.55-2.83) | 2.51  (1.29-4.86) | 2.00  (0.93-4.30) | 0.64  (0.24-1.70) | 0.51  (0.16-1.57) | 2.12  (0.97-4.64) | 1.11 (0.45-2.7) |
| Other ethnic background | 1.67  (1.08-2.59) | 1.40  (0.82-2.38) | 2.39  (1.76-3.25) | 1.84  (1.25-2.74) | 2.23  (1.68-2.97) | 1.45  (1.02-2.07) | 2.77  (2.06-3.73) | 1.78  (1.21-2.61) | 2.60  (1.89-3.57) | 1.68  (1.12-2.50) |

*Note*. *OR* odds ratio, *CI* confidence interval, *FA* fully adjusted, *WB* White and Black*.* ^a^ Fully adjusted model adjusts for all sociodemographic and clinical variables in the study. Broad confidence intervals suggest reduced power, due to small observations in the cell.

Table S6. Stratified analysis (crude and fully adjusted) of the relationship between ethnicity and involuntary admission under the MHA by homelessness

| Ethnicity | Not homeless | | Homeless | |
| --- | --- | --- | --- | --- |
|  | Crude OR  (95% CI) | FA OR^a^ (95% CI) | Crude OR  (95% CI) | FA OR^a^ (95% CI) |
| White British | (ref) | (ref) | (ref) | (ref) |
| White Irish | 1.19  (0.95-1.50) | 1.03  (0.80-1.34) | 1.32  (0.79-2.22) | 1.05  (0.59-1.86) |
| Other White background | 2.03  (1.80-2.29) | 1.46  (1.25-1.70) | 4.57  (3.54-5.91) | 1.92  (1.41-2.61) |
| Black African | 3.08  (2.76-3.43) | 1.72  (1.48-1.99) | 7.34  (5.47-9.84) | 2.83  (2.01-3.99) |
| Black Caribbean | 2.98  (2.61-3.41) | 1.63  (1.39-1.90) | 4.96  (3.26-7.54) | 1.97  (1.21-2.22) |
| Black British | 2.98  (2.66-3.33) | 1.75  (1.54-2.00) | 3.87  (2.84-5.28) | 1.79  (1.25-2.56) |
| Asian Bangladeshi | 2.81  (1.78-4.44) | 1.70  (1.02-2.83) | 3.56  (1.12-11.29) | 1.67  (0.44-6.37) |
| Asian Indian | 1.90  (1.46-2.48) | 1.45  (1.08-1.95) | 1.40  (0.59-3.33) | 1.37  (0.52-3.60) |
| Asian Pakistani | 2.10  (1.51-2.93) | 1.58  (1.09-2.31) | 3.91  (1.21-12.61) | 2.46  (0.68-8.98) |
| Asian Chinese | 2.93  (2.03-4.24) | 2.04  (1.34-3.09) | 13.05  (4.48-38.03) | 6.23  (1.90-20.49) |
| Asian British | 1.96  (1.64-2.33) | 1.46  (1.19-1.80) | 3.67  (2.24-6.01) | 1.72  (0.97-3.06) |
| Mixed WB African | 1.56  (0.95-2.55) | 0.98  (0.57-1.70) | 0.75  (0.10-5.79) | 0.66  (0.08-5.39) |
| Mixed WB Caribbean | 1.46  (1.07-1.99) | 1.10  (0.78-1.55) | 1.19  (0.47-3.06) | 1.08  (0.36-3.21) |
| Other Mixed background | 1.47  (1.04-2.10) | 1.12  (0.76-1.66) | 2.85  (1.21-6.72) | 1.98  (0.77-5.09) |
| Other ethnic background | 2.01  (1.75-2.32) | 1.58  (1.33-1.87) | 5.16  (3.53-7.53) | 2.60  (1.68-4.04) |

*Note*. *OR* odds ratio, *CI* confidence interval, *FA* fully adjusted, *WB* White and Black*.* ^a^ Fully adjusted model adjusts for all sociodemographic and clinical variables in the study. Broad confidence intervals suggest reduced power, due to small observations in the cell.

Table S7. Stratified analysis (crude and fully adjusted) of the relationship between ethnicity and involuntary admission under the MHA by migrant status

|  | No Migrant Status | | Migrant status | |
| --- | --- | --- | --- | --- |
| Ethnicity | Crude ORs (95% CI) | FA^a^ ORs  (95% CI) | Crude ORs (95% CI) | FA^a^ ORs  (95% CI) |
| White British | (ref) | (ref) | (ref) | (ref) |
| White Irish | 1.38  (0.88-2.16) | 1.29  (0.75-2.20) | 0.97  (0.60-1.56) | 1.05  (0.62-1.77) |
| Other White background | 1.61  (1.07-2.44) | 1.26  (0.77-2.06) | 2.35  (1.58-3.49) | 1.72  (1.10-2.67) |
| Black African | 6.41  (4.48-9.16) | 2.50  (1.64-3.81) | 3.38  (2.27-5.03) | 2.04  (1.31-3.19) |
| Black Caribbean | 3.21  (2.46-4.19) | 1.55  (1.12-2.14) | 2.99  (1.93-4.61) | 1.68  (1.04-2.73) |
| Black British | 3.27  (2.82-3.78) | 1.65  (1.37-1.99) | 3.30  (2.08-5.23) | 2.20  (1.31-3.67) |
| Asian Bangladeshi | 3.68  (1.49-9.07) | 2.67  (0.81-8.79) | 2.60  (1.30-5.23) | 1.73  (0.79-3.79) |
| Asian Indian | 1.34  (0.77-2.32) | 1.09  (0.56-2.14) | 1.65  (0.96-2.83) | 1.58  (0.86-2.88) |
| Asian Pakistani | 2.60  (1.33-5.10) | 1.04  (0.47-2.28) | 1.65  (1.48-4.83) | 2.11  (1.09-4.10) |
| Asian Chinese | 2.04  (0.51-8.18) | 1.20  (0.24-6.14) | 3.24  (1.86-5.66) | 2.34  (1.26-4.38) |
| Asian British | 2.34  (1.50-3.67) | 1.33  (0.78-2.26) | 1.95  (1.26-3.01) | 1.59  (0.98-2.59) |
| Mixed WB African | 1.60 (0.74-3.46) | 0.83  (0.33-2.10) | 1.88  (0.79-4.45) | 1.20  (0.45-3.15) |
| Mixed WB Caribbean | 1.47  (0.98-2.19) | 1.22  (0.75-1.98) | 1.30  (0.43-3.91) | 0.91  (0.28-2.99) |
| Other Mixed background | 2.09  (1.26-3.47) | 1.48  (0.80-2.71) | 1.09  (0.47-2.53) | 0.90  (0.35-2.31) |
| Other ethnic background | 2.00  (1.25-3.22) | 1.19  (0.68-2.07) | 2.65  (1.74-4.03) | 2.17  (1.36-3.46) |

*Note*. *OR* odds ratio, *CI* confidence interval, *FA* fully adjusted, *WB* White and Black*.* ^a^ Fully adjusted model adjusts for all sociodemographic and clinical variables in the study. Broad confidence intervals suggest reduced power, due to small observations in the cell.
